# DYNAMO-A: A generic simulation model coupling crop growth and disease epidemic

**DOI:** 10.1371/journal.pone.0321261

**Published:** 2025-04-24

**Authors:** Laetitia Willocquet, Simone Bregaglio, Roberto Ferrise, KH Kim, Serge Savary

**Affiliations:** 1 Département Santé des Plantes et Environnement, INRAE, Toulouse, France; 2 Department of Agrometeorology, G. B. Pant University of Agriculture and Technology, Pantnagar, India; 3 Council for Agricultural Research and Economics (CREA) – Research Centre for Agriculture and Environment, Bologna, Italy; 4 Department of Agriculture, Food, Environment and Forestry, University of Florence, Florence, Italy; 5 Department of Agricultural Biotechnology, Seoul National University, Seoul, Korea; 6 Department of Plant Pathology, University of California-Davis, Davis, United States of America; 7 Department of Plant Pathology, ICAR-Indian Agricultural Research Institute, New Delhi, India; 8 Department of Plant Pathology, G. B. Pant University of Agriculture and Technology, Pantnagar, India; Indian Agricultural Research Institute, INDIA

## Abstract

Very few dynamic simulation models truly involve explicit, quantitative, two-way couplings of epidemiological and agrophysiological processes. Our aim is to develop a generic, transparent and simple, coupled disease-crop model, DYNAMO-A, where a polycyclic epidemic develops within the canopy of an annual crop. DYNAMO-A builds upon existing models, RICEPEST and WHEATPEST, respectively designed as crop loss simulation platforms for rice and wheat, and the generic model GENEPEST, which was designed for further crop-specific development and educational purposes. Two intertwined components constitute DYNAMO-A: (1) an agrophysiological component simulates crop growth, which alters the carrying capacity of epidemics; and (2) an epidemiological component simulates epidemics, which affect crop growth through damage mechanisms. Analyses using DYNAMO-A consider different simulation scenarios according to the pathogen lifestyle (trophism) and production situations. First, scenarios consider a biotrophic ideotype which is a light stealer and assimilate diverter, and a necrotrophic ideotype which is a light stealer and an accelerator of leaf senescence. Second, scenarios consider two production situations (favourable or less favourable), i.e., two contexts leading to differing attainable (un-injured) crop yields (good or average). Epidemics caused by a biotrophic pathogen reduce the green leaf area and diverts plant assimilates to the pathogen tissues, resulting in a decrease in yield. In epidemics caused by a necrotrophic pathogen, both diseased and green leaf areas are reduced because of disease-induced senescence, resulting also in yield loss. Overall, at a given level of disease epidemic, absolute yield losses are higher with a biotrophic pathogen in a more favourable production situation, whereas yield losses to a necrotrophic pathogen tend to be similar irrespective of production situations. Our results concur with previous studies, both field-experiment and model-based, on several crop-disease systems. Future modelling with DYNAMO-based models should enable interdisciplinary research addressing plant disease impacts on current and future agricultural production.

## 1. Introduction

Plant disease epidemics result from dynamic interactions between pathogen and host populations. On the one hand, epidemics are constrained by the host carrying capacity which varies over time [1]. Epidemics are further influenced by climate and microclimate [2], host spatial structure [3–5], and host tissue susceptibility [6]. On the other hand, host physiological processes are impaired by plant disease according to damage mechanisms [7–10], which can broadly be classified as Radiation Use Efficiency (RUE)-reducing, or radiation interception (RI)-reducing [11]. Among them, light stealing (a decrease in green leaf area), accelerated leaf senescence (or defoliation), and assimilate diversion are the most important damage mechanisms, especially for non-systemic diseases affecting the crop canopy [12, 13]. These damage mechanisms are strongly associated with the trophism of pathogens affecting crop canopies: while biotrophic pathogens are generally light stealers and assimilate diverters, necrotrophic pathogens are generally light stealers and leaf senescence accelerators [12, 13]. This difference has important implications on the net impact of epidemics on crop growth and yield losses [10].

Given the massive impacts of disease on crop production [14, 15], modelling crop losses to diseases (and other yield-reducing factors) is necessary for a number of reasons: a better understanding of the underlying processes is required for progress in limiting such impacts; the necessity to prioritise the impacts of diseases for their management [16]; the difference of disease impacts depends on production situations, represented by the un-injured crop yield level (attainable yield; [17]); and the behaviour of crop-disease systems under climate change is essentially unknown, despite some strong hypotheses based on field data [18]. With crop losses from diseases and insect pests ranging from 10 to 40% [19], the bulk of the yield estimates generated today through crop models [20] is overestimated by a factor of 1.1 to 1.5, because these yield-reducing factors (pathogens, pests, and weeds) are not accounted for. In times when we must be able to inform on what the future of agricultural performances could be [18], such a gap, and such an uncertainty about this gap, are inacceptable. While crop loss modelling needs experimental data for model parameterisation and evaluation, the international community is in dire need of adequate crop loss modelling capacity: this is because crop loss data are so rare and patchy [15]. A collective, interdisciplinary effort is both urgent and necessary [19].

Different approaches have been implemented to model plant disease epidemics and their impacts on crops, depending on the relative emphasis on the epidemiological or on the physiological processes. Epidemiological models have extensively used the SEIR (Susceptible-Exposed-Infectious-Removed) structure to represent polycyclic epidemics on the aerial parts of plant tissues [21–23]. In these models, the dynamic of the host carrying capacity is generally represented in a synthetic way, for example as a logistic growth (e.g., [24–29]). As for agrophysiological models, disease effects generally are incorporated as damage mechanisms. The approach has been used to derive yield loss as the difference between simulated attainable yield (with no disease) and simulated actual yield (with disease; [30, 31]). In such models, the dynamics of disease intensity are generally represented as model drivers, similarly to weather variables [22].

Coupled crop-disease models, i.e., models where epidemiological and agrophysiological processes are truly intertwined, have been in comparison much less developed. Fully coupled models account for the epidemiological and physiological underlying processes with different degrees of detail.

A few coupled models have represented epidemics and crop growth in a synthetic way, according to logistic functions. For example, a generic model was designed to couple epidemic dynamics with leaf area dynamics [1]. In this model, the dynamics of the green leaf area is reduced according to disease severity (i.e., light stealing damage mechanism), while disease progress is limited by the host carrying capacity (green leaf area). Another generic coupled model further accounted for competition between diseases caused by necrotrophic and biotrophic pathogens, and considered two types of damage mechanisms: RI-reduction and RUE-reduction [10].

Detailed models coupling epidemics and crop growth have also been developed. Several coupled models have considered specific pathosystems such as potato early blight [32], groundnut rust [33], wheat septoria nodorum blotch [34], potato late blight [35, 36], rice blast [37–39], and wheat leaf rust [37,40]. The latter two studies on leaf rust did not consider assimilate diversion, therefore leading to an underestimation of the impact of disease on crop growth and of yield loss. Generic coupled models which account for light stealing only [41], or light stealing and reduction in photosynthesis efficiency [37] have been developed. To the best of our knowledge, no generic coupled model exists which accounts for light stealing, assimilate diversion, accelerated leaf senescence, and RUE reduction.

A first objective is to develop DYNAMO-A as a generic, shareable, transparent, and simple model structure with an explicit two-way coupling of epidemiological processes (disease increase through infection, latency, infectious, and post-infectious stages of infected plant tissues limited by host carrying capacity) and agrophysiological processes (damage mechanisms from disease: light stealing, acceleration of leaf senescence, assimilate diversion, and RUE reduction). DYNAMO-A builds upon existing modelling structures, RICEPEST [42] and WHEATPEST [43], respectively designed as crop loss simulation platforms for rice and wheat, and GENEPEST [44], a generic model designed for further crop-specific development and educational purposes. DYNAMO-A considers polycyclic epidemics affecting the canopy of an annual crop. A second objective is to analyse the dynamic relationships between epidemics and crop growth with DYNAMO-A according to pathogen trophism and production situation. These two objectives are part of a collective effort (the Global Plant Health Assessment) where a community of scientists worldwide attempts assessing the state and the future of plant health globally (https://sites.google.com/view/global-plant-health-assessment/home).

## 2. Materials and methods

### 2.1. Model structure and hypotheses

This work focuses on processes leading to host-pathogen interactions at the population level, and their effects on the behaviour of pathogen-crop systems. The effects of either climate, or specific crop management practices, or factors inherent to the specificity of host-pathogen interactions (such as host plant resistance), or again the biological environment of host-pathogen interactions (e.g., [45]) are not addressed, in order to retain model simplicity.

**Fig 1:**
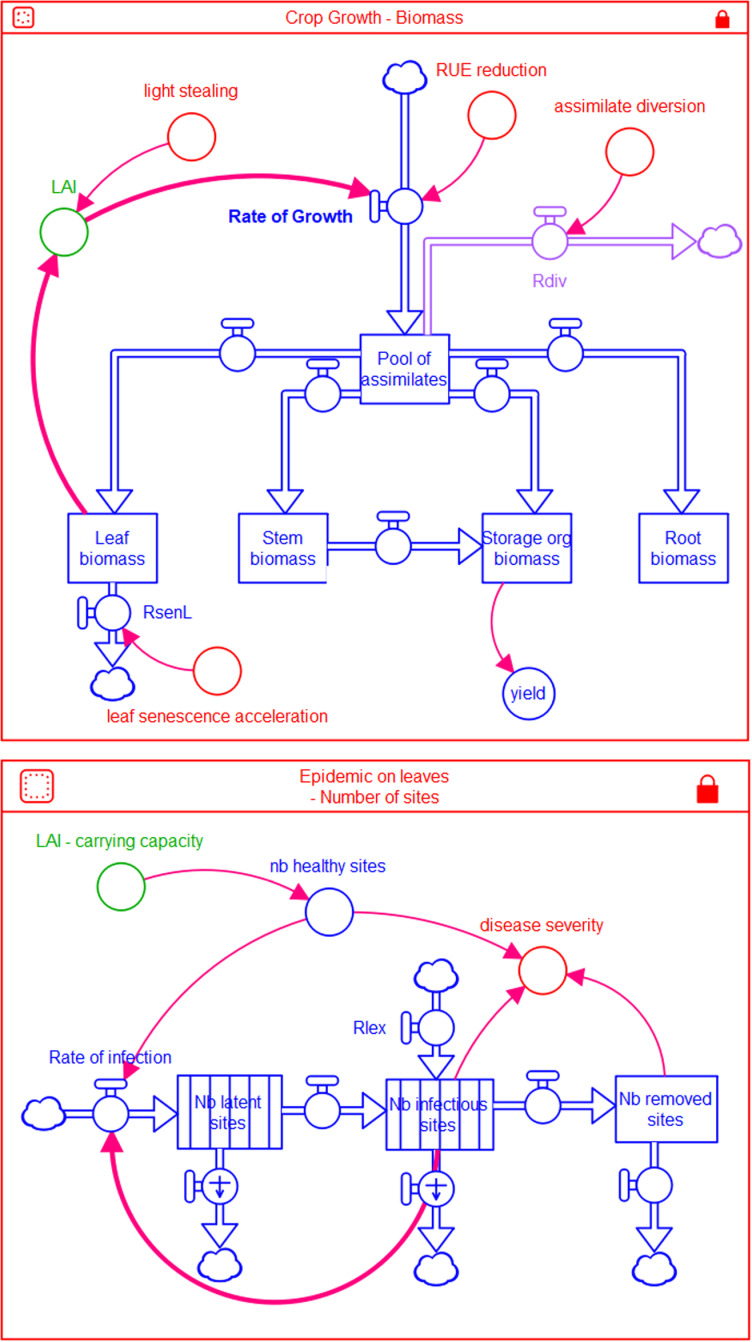
Simplified flowchart of DYNAMO-A, with crop growth and epidemic coupled. Coupling from epidemic to crop growth: Red variables in the crop growth component represent the damage mechanisms modelled, which depend on disease severity, in turn computed in the epidemic component. Coupling from crop growth to epidemic: the LAI computed in the crop growth component is used to compute the number of healthy sites available for infection in the epidemic component. Symbols (state variables: rectangles, rates: valves, flows: double arrows; numerical relations: simple arrows; parameters (fixed or dynamically calculated) and driving functions: circles) used for variables are derived from Forrester [102]. Bold arrows highlight the feedback loops in crop growth and epidemic models. Rdiv: rate of assimilate diversion; rlex: rate of lesion expansion; rsenL: rate of leaf senescence.

The overall model organisation is shown in Fig 1, and the model flowchart is displayed in S1 Fig. Variables used in the model are described (acronyms, acronym meanings and variable computation, components of the model to which the variable belongs, variable dimensions, and variable units) in Table 1 and in S1 Table. The model code is provided in S1 Code.

**Table 1:** Description of the key variables used in DYNAMO-A.

Acronym	Meaning/computation	Dimension	Unit
InfS	Number of infectious sites	[N]	NSites
LatS	Number of latent sites	[N]	NSites
LeafB	Biomass of leaves	[M]	g
Pool	Pool of assimilates	[M]	g
RemS	Number of removed sites	[N]	N
RootB	Biomass of roots	[M]	g
StemB	Biomass of stems and leaf sheaths	[M]	g
StorB	Biomass of storage organs	[M]	g
RGrowth	Rate of crop growth	[M.T^-1^]	g.day^-1^
Rdiv	Rate of diversion of assimilates	[M.T^-1^]	g.day^-1^
RI	Rate of infection	[N.T^-1^]	NSites.day^-1^
RPI	Rate of primary infection	[N.T^-1^]	NSites.day^-1^
RLEX	Rate of lesion expansion	[N.T^-1^]	NSites.day^-1^
RSenL	Rate of leaf biomass senescence	[M.T^-1^]	g.day^-1^
RSenS	Rate of senescence on sites	[N.T^-1^]	NSites.day^-1^
β	Ratio of virtual over visible lesion area	[1]	–
DVS	Development stage – depends on STEMP	[1]	–
FNG	Fraction of non-green area in infectious or removed sites	[1]	–
FS	Fraction of sporulating area in infectious sites	[1]	–
i	Infectious period	[T]	day
k	Coefficient of light extinction	[1]	–
p	Latency period	[T]	day
RAD	Daily radiation	[E.T^-1^]	MJ.day^-1^
Rc	Intrinsic rate of disease increase	[T^-1^]	day^-1^
RFRUE	Reduction factor for RUE reducer	[1]	–
rrdiv	Relative rate of diversion of assimilates	[M. L^-2^.T^-1^]	g.m^-2^.day^-1^
rrds	Relative rate of disease-induced senescence	[T^-1^]	day^-1^
rrsen	Relative rate of physiological leaf senescence	[T^-1^]	day^-1^
RUE	Radiation Use Efficiency	[M.E^-1^]	g.MJ^-1^
SizeS	Size of a site	[L^2^]	m^2^
SLA	Specific Leaf Area	[L^2^.M^-1^]	m^2^.g^-1^
SMax	Maximum number of sites which can be occupied on 1 m^2^ of leaves	[N.L^-2^]	NSites.m^-2^
CORF	Correction factor	[1]	–
H	Number of healthy sites	[N]	NSites
IRSi	Number of infectious and removed sites	[N]	NSites
LAI	Non senesced, healthy and diseased LAI	[L^2^]	m^2^
gLAI	Green LAI	[L^2^]	m^2^
OccS	Number of occupied sites	[N]	NSites
phLAI	Photosynthetically active LAI	[L^2^]	m^2^
rrsenD	Relative rate of disease-induced senescence	[T^-1^]	day^-1^
s	Fraction of LAI covered by sporulating pustules	[1]	–
sev	Disease severity	[1]	–
TotSi	Maximum number of sites which can be occupied on 1 m^2^ of ground	[N]	NSites

Dimensions and units consider a system of 1 m^2^ of crop.

#### 2.1.1. Building models: GENEPEST and EPIDEM.

The model involves two inter-connected components, each representing distinct processes: crop growth and plant disease epidemic. Many processes of the crop growth component vary according to phenology, which is also simulated. The model time step in one day, and the system considered is 1 m^2^ of a standing (annual) crop. The model was implemented with the STELLA software (https://www.iseesystems.com/).

The first component accounts for the physiological processes that are involved in crop growth and yield build-up. This component simulates the dynamics of biomass growth of the different organs according to crop development stages. This component is derived from GENEPEST, a generic agrophysiological model which was developed for annual crops [44], such as rice [42] and wheat [43].

Development is the process through which an organism transits from a given physiological stage to a succeeding one (e.g., for a plant, from seed to seedling, from seedling to a vegetative stage, from vegetative to flowering, and so on till senescence; [46]). Here, the development stage (*DVS*) of a given crop is scaled from 0 (emergence) to 1 (flowering) and 2 (crop physiological maturity; [46]). Plant development is assumed to depend linearly on thermal accumulation [47, 48]. In an annual crop plant, *DVS* is determined by the temperature threshold for crop development (*TBASE*), the sum of temperature required to reach the flowering stage (*TFLOW*), and the sum of temperature required to reach physiological maturity (*TMAT*).

Crop growth, i.e., the dynamics of biomass of plant tissues, is governed by photosynthesis, leading to the daily accumulation of a pool of assimilates. A key equation of the agrophysiological component is the rate of crop growth, written as (e.g., [48, 49]):


$$RGrowth=RAD\times RUE\times e^{-k\times LAI}$$ (1)

Where *RAD* is the daily global radiation, *RUE* is the radiation use efficiency, *LAI* is the leaf area index, and *k* is Beer’s coefficient of light extinction (Table 1). *LAI* is computed from leaf biomass, and represents the non-senesced leaf area, which can be healthy or diseased.

The pool of carbohydrates generated by *RGrowth* (Equation 1) is partitioned daily to the different plant organs: roots (*RootB*), leaves (*LeafB*), stems (*StemB*), and storage organs (*StorB*). The partitioning towards organs depends in turn on development stage. From plant emergence until the seedling stage, the bulk of carbohydrates are directed towards roots and leaves. During the vegetative stage, partitioning of carbohydrates towards roots tapers off, while partitioning towards stems increases. At anthesis, sinks associated with storage organs are formed, and start accumulating biomass through daily increments. As crop maturity approaches, the leaf biomass progressively declines as physiological senescence and leaf shedding (in dicots) or drying up (monocots) take place. The modelling structure described here concentrates on cereals. We therefore refer to leaf blades, leaf senescence, and grains in the following description. The rate of physiological leaf [blade] senescence is written as:


RSenL=rrsen×LeafB (2)

Where *rrsen* is the relative rate of physiological leaf senescence and *LeafB* is the biomass of non-senesced, healthy and diseased leaves [leaf blades].

Crop ripening also leads to a fraction of the accumulated stem biomass being translocated towards storage organs. Starch translocation towards storage organs thus contributes to the final crop yield [42,43,46]. The final value of *StorB* is used to represent the yield.

The second component of the model represents the dynamics of an epidemic on the foliage. This component is derived from the epidemiological model developed by Zadoks (1971; [23]), and displayed as EPIDEM in Savary and Willocquet (2014; [44]). Following Savary et al [28, 29], additional model features entail the consideration of crop growth and lesion expansion. This component simulates the dynamics of sites, which can be healthy, latent (after infection has occurred), then become infectious, and finally removed from the epidemiological processes. Latent (*LatS*), infectious (*InfS*) and removed (*RemS*) sites belong to the category of occupied sites (*OccS*). Infection leading to the increase in number of latent sites originates from two sources: primary inoculum (leading to primary infections), and infectious sites (leading to secondary infections, and featuring the feed-back loop of a disease cycle in polycyclic epidemics). Infectious sites can also originate from lesion expansion. Epidemics are limited by the carrying capacity of the host population, represented by the correction factor, *CORF*, which affects both infection and lesion expansion. *CORF* is expressed as the fraction of healthy sites remaining available for infection. A key equation of the epidemiological component is the rate of infection, written as:


$$RI=RPI+\left(Rc\times InfS\times CORF\right)$$ (3)

Where *RPI* is the rate of primary infections, *Rc* is the intrinsic rate of disease increase [50], also referred to as *DMFR* (daily multiplication factor; [23]); and *InfS* is the number of infectious sites.

An important departure from classical epidemiological models is the absence of a state variable corresponding to the number of healthy sites, *H*. Instead, this quantity is derived from the number of diseased sites and from the *LAI*, as described in the following section.

#### 2.1.2 Coupling host to disease dynamics: carrying capacity of epidemics.

This coupling concerns the carrying capacity that the host provides for a pathogen according to the *LAI*, the number of healthy sites (*H*), and physiological senescence.

The carrying capacity of an epidemic [in the case of a disease affecting leaf blades] is derived from leaf biomass as follows: *LeafB* allows calculating the *LAI* (eq. 4), that is, the area of non-senesced, healthy and diseased leaves [i.e., the area of leaf blades]; the daily *LAI* value allows in turn deriving *TotSi* (eq. 5), that is, the carrying capacity expressed as the total number of sites in a crop established on 1 m^2^ of ground (the system size); the correction factor is then computed as the fraction of non-occupied sites (eq. 6). The sequence *LeafB* - > *LAI* - > *TOTSI* - > *CORF* is therefore derived as follows:


LAI=LeafB×SLA (4)

TotSi=LAI×SMax (5)

$$CORF=1-\left(OccS/TotSi\right)$$ (6)

OccS=LatS+InfS+RemS (7)

Where *SLA* is the specific leaf area and *SMax* is the maximum number of sites which can be occupied in 1 m^2^ of host [leaf blade] tissue.

The number of healthy sites can in turn be derived from *OccS* and *totSi* as:


H=TotSi−OccS (8)

The physiological senescence occurring after flowering results into an overall loss of sites, under the hypothesis that the relative rate of senescence of sites is independent from the site category (healthy, latent, infectious, or removed). The rate of senescence is therefore written for all sites categories as:


RsenS=rrsen×S (9)

Where *S* is the number of sites of a given category (healthy, latent, infectious, or removed).

#### 2.1.3. Coupling disease to host dynamics: the pathogen damage mechanisms.

Four damage mechanisms are considered in the model. These mechanisms represent the main ways (although not all) through which a yield-reducer affects the physiology of a host crop: light stealing, assimilate diversion, leaf senescence acceleration (defoliation), and RUE reducing [7–9].

**Light stealers** typically decrease green leaf [blade] areas, occupied by lesions (spots, pustules) which belong to infectious or removed sites. In order to account for this damage mechanism, it is convenient to introduce two quantitative features of the disease: the size (area) of a site (*SizeS*) and the fraction of non-green area in infectious or removed sites (*FNG*). This fraction is not always equal to 1. For example, it is estimated at 0.37 in the case of cereal rusts [51]. Disease severity (*sev*), defined here as the fraction of *LAI* which is not green (e.g., covered by spots or pustules, or by a chlorotic area surrounding them), can then be written as:


sev=SizeS×FNG×IRSi/LAI (10)

with


IRSi=InfS+RemS (11)

*sev* allows computing the green *LAI* (*gLAI*), which can be useful to characterise the performance of a crop:


$$gLAI=LAI\times \left(1-sev\right)$$ (12)

The model further accounts for pathogens associated with “virtual lesions” [52], whereby the green area surrounding the pustule does not photosynthesise, and therefore does not contribute to *Rgrowth*. This decrease in photosynthetic area is incorporated in equation (1), which becomes:


$$RGrowth=RAD\times RUE\times \left(1-e^{-k\times phLAI}\right)$$ (13)

With


$$phLAI=LAI\times {\left(1-sev\right)}^{\beta }$$ (14)

where *phLAI* is the photosynthetically active *LAI*, and *β* is a parameter representing the ratio of the virtual lesion area over the pustule area.

**Assimilate diversion** is incorporated as an outflow from the pool of assimilates which is proportional to the area covered by sporulating pustules:


Rdiv=rrdiv×s×LAI (15)

With


s=FS×InfS/TotSi (16)

where *rrdiv* is the relative rate of assimilate diversion, *s* is the fraction of *LAI* covered by sporulating pustules, and *FS* is the fraction of the area of an infectious site covered by a sporulating pustule.

**RUE-reducing** is incorporated in the model as a reduction factor for *RUE*, which is proportional to disease severity, and equation (13) becomes:


$$RGrowth=RAD\times RUE\times \left(1-e^{-k\times phLAI}\right)\times \left(1-\left(RFRUE\times sev\right)\right)$$ (17)

Where *RFRUE* is the reduction factor for RUE reducer, that is, the fraction of *RUE* reduced per unit of disease severity.

**Leaf senescence acceleration (defoliation)** is represented in the model under three hypotheses:

- the relative rate of disease-induced senescence is proportional to disease severity;

- interactions occur between physiological senescence and disease-induced senescence: an area affected from one process cannot be affected by the other;

- only senescence of leaf blades is considered here.

The effect of disease-induced senescence on leaf biomass is incorporated, and equation (2) becomes:


$$RSenl=\left(rrsen+rrsenD-\left(rrsen\times rrsenD\right)\right)\times LeafB$$ (18)

With


rrsenD=rrds×sev (19)

where *rrds* is the relative rate of disease-induced senescence, per disease severity unit.

The effect of disease-induced senescence on latent, infectious, and removed sites is incorporated, and equation (9) becomes:


$$RsenS=\left(rrsen+rrsenD-\left(rrsen\times rrsenD\right)\right)\times S$$ (20)

Physiological and disease-induced leaf senescence therefore affect the leaf biomass, and in turn reduce the number of sites in the epidemiological model component.

The relative rate of senescence for infectious sites was computed separately, although with the same equation, to avoid trouble shooting of initial circularity in the STELLA programme.

### 2.2. Model parameters and simulation runs

The focus of the current work is to investigate the reciprocal effects of crop growth and epidemic dynamics. The effects of additional factors (e.g., environmental variability) on these interactions were therefore not considered in the analyses conducted with DYNAMO-A. Simulations start at seedling stage (time =  0; *DVS* =  0.33, 2–3 expanded leaves) and stop at physiological maturity (*DVS* =  2). Parameter values of the model are listed in Table 2. A first group of parameters were set at identical values for all simulations and are described below.

**Table 2:** Parameter values used for simulation of scenarios with DYNAMO-A.

Parameters^a^	Values^b^			References	
**Parameters identical in all scenarios**					
β	1			[52]	
i	20			[28,29,53,54,55]	
k	0.6			[44]	
onset	10			[28]	
p	10			[28,29,53,54,55]	
RAD	17			[44]	
RFRUE	0			NA	
rrlex	0			NA	
SizeS	10^-5^			[56,57]	
TBASE	8			[44]	
TFLOW	1,500			[44]	
TMAT	2,000			[44]	
TMAX	30			[44]	
TMIN	24			[44]	
**Parameters depending on trophism ideotype**					
	Biotroph ideotype		Necrotroph ideotype		
FNG	0.5		1	[51]	
FS	0.5		1	[51]	
rrds	0		0.1	[33,69]	
SMax	10^5^		5.10^4^	[28,29,56]	
**Parameters depending on conditions of crop growth** [**and trophism ideotype for Rc and rrdiv)**					
	GPS	APS	GPS	APS	
Rc	1	0.66	1		[28,29,68]
rrdiv	5	3.33	0		[43,65,66]
Priminoc	200	120	100	60	Current work
RUE	1.2	0.8	1.2	0.8	[49,59]

^a^See Table 1 for meaning of acronyms.

^b^GPS: crop grown under a good production situation; APS: crop grown under an average production situation; Priminoc was set so that disease severity at day 21 is 0.05%; See Table 1 for units.

Climate drivers, development stage and agrophysiological parameters have been set as in GENECROP [44]. The parameter and driving function values correspond to a tropical annual crop such as rice grown under rainy season weather conditions. Climate drivers, i.e., temperature and radiation, are represented in the simplest way and are kept constant over simulations. Under the pre-set temperature values (*TMIN* = 24°C, *TMAX* = 30°C; Table 2) and development stage parameters (*TFLOW* = 1,500°C.day; *TMAT* =  2,000°C.day; Table 2), physiological maturity (*DVS* =  2) is achieved at time (day) 90.

Latency (*p*) and infectious periods (*i*) were set to 10 and 20 days, respectively. These values are fairly common for foliar diseases under optimal or sub-optimal conditions [28,29,53]. For instance, *p* and *i* values are in the range of 5–10 and 15–25 days, respectively, in the case of rice leaf blast [54]. These parameters are in the range of 8–12 and 14–37 days, respectively for *p* and *i*, in the case of septoria tritici blotch on winter wheat [55].

The area of a site (*SizeS*) was set to 10 mm^2^, which corresponds to medium size lesions or to the initial area of lesions prior to their expansion [56], as for example in the case of brown spot of rice [57].

The time of disease onset (*onset*) was set at 10 days to represent early primary infections and epidemic onset. The rate of primary infections (*Priminoc*) is very poorly documented in the literature. Furthermore, the effect of primary infections on epidemics was not studied in the present work. It was therefore decided to start all epidemics with the same disease severity level, and *Priminoc* was set so that a disease severity of 0.05% is reached at day 21.

### 2.3. Simulations of scenarios according to pathogen trophism and crop production situations

A limited number of scenarios were considered in order to examine the behaviour of the simulated crop-epidemic system. These scenarios involved two ideotypes of pathogens according to their trophism and associated damage mechanisms, and two conditions of crop growth (production situations). The scenario-specific parameters are listed in Table 2.

Two production situations [17,58] of a crop were considered, good and average, and were represented by setting *RUE* values at 1.2, and 0.8 g MJ^-1^, respectively. An *RUE* of 1.2 g MJ^-1^ represents favourable crop growth conditions [49,59]. These *RUE* values led to attainable (non-injured) yields of 684 and 372 g m^-2^, respectively (that is, 6.84 and 3.72 t ha^-1^).

We first considered the ideotype of a biotrophic pathogen feeding on living tissues such as rust-causing pathogens. Biotrophic pathogens are light stealers and assimilate diverters. We did not consider disease-induced senescence caused by biotrophic pathogens, which is an assumption that may hold for most cereal rusts [60], but does not for other rusts, e.g., as in coffee rust caused by *Hemileia vastatrix* [61], or in soybean rust caused by *Phakopsora pachyrizi* [62, 63] or grapevine rust caused by *Neophysopella tropicalis* [63].

We considered a biotrophic pathogen for which an infected site consists of a pustule and its surrounding fungus-colonised area. We further considered that half of an infected site area is covered by a pustule (*FNG* = 0.5 and *FS* = 0.5), which is close to values estimated for biotrophs like rusts [51]. Such a value of *FNG* corresponds to the hypothesis that, in the case of biotrophic pathogens, mycelial structures occupy tissues that lie beyond the pustule [51], but belong to the same lesion. We further considered that any site in the canopy may be occupied (diseased), so that *SMax*, the maximum number of sites which can be occupied in 1 m^2^ of host, was set as the inverse of *SizeS*.

Diversion of assimilates from biotrophic pathogens such as rusts is mainly used for spore production [64]. The relative rate of assimilates diversion in the scenario involving a crop grown under a good production situation was set to 5 g. m^-2^, a value estimated in the case of wheat leaf rust [43]. Sporulation intensity of biotrophs may increase with nitrogen inputs in the crop, as was shown in the case of wheat leaf rust [65], and barley powdery mildew [66]. This pattern may be due to the positive effect of nitrogen input on the rate of photosynthesis (e.g., [67]), leading to an increased amount of assimilates produced, which can in turn be diverted by the pathogen. As no quantitative relationship between the rate of sporulation and the rate of photosynthesis could be retrieved from the literature, the simplest hypothesis was forwarded to represent this effect in DYNAMO-A, that is, *rrdiv* was assumed to be proportional to *RUE*, resulting in a value of *rrdiv* of 3.33 g day^-1^ m^-2^ when *RUE* =  0.8 g MJ^-1^.

Values of the intrinsic rate of disease increase (*Rc*) are seldom reported. *Rc* values for susceptible varieties grown under conditions favourable to epidemics were estimated at 1.14 day^-1^ for rice leaf blast and 0.61 day^-1^ for rice brown spot [28], 1.47 day^-1^ for wheat leaf rust and 1.17 day^-1^ for wheat septoria tritici blotch [29], and 1.1 day^-1^ for wheat stripe rust [68]. A rounded value of 1.0 day^-1^ was used for the simulated scenario under good production situation. Because the *Rc* accounts for the daily spore production, *Rc* was made proportional to *rrdiv* and set to 0.66 day^-1^ under the scenario of average production situation.

We then considered the ideotype of a necrotroph pathogen. Necrotroph pathogens are light stealers and leaf senescence accelerators. For this second pathogen ideotype, we considered that the entire site area is covered by a lesion (*FNG* = 1). The fraction of a leaf area covered by lesions of necrotrophic pathogens on field crops seldom exceeds 50% [56]. We therefore further hypothesized that 50% of the sites can be occupied by the ideotype of necrotrophic pathogen, and *SMax* was set as 1/(2 x *SizeS*). *rrds* values were estimated at 0.076 day^-1^ for rice sheath blight [69], and decreased from 0.24 to 0.08 day^-1^ when groundnut cercospora severities increased from 10 to 50% [33]. A rounded *rrds* value of 0.1 day^-1^ was set for the necrotroph ideotype in both scenarios of production situation.

Simulated dynamics of variables corresponding to both ideotypes under both production situations were displayed: (1) the numbers of healthy, latent, infectious, and removed sites; (2) the LAI and disease severity (expressed as the percent of diseased sites); and (3) the pool of assimilates and the rate of assimilate diversion in the case of biotrophs.

Synthetic variables resulting from each simulated run were computed: the actual (i.e., injured, diseased) crop yield (*Y*); the attainable (non-injured, healthy) yield (*Ya*); yield loss (*YL*, the difference between the attainable, *Ya*, and the actual, *Y*, yields) and relative yield losses (*RYL*, the percentage of yield lost to diseases, relative to the attainable yield); the accumulated biomass of assimilates diverted for the biotroph ideotype, and the accumulated biomass of leaves senesced from disease for the necrotroph ideotype.

## 3. Results

### 3.1. Simulated epidemics and crop growth for a biotroph in two production situations

**Fig 2:**
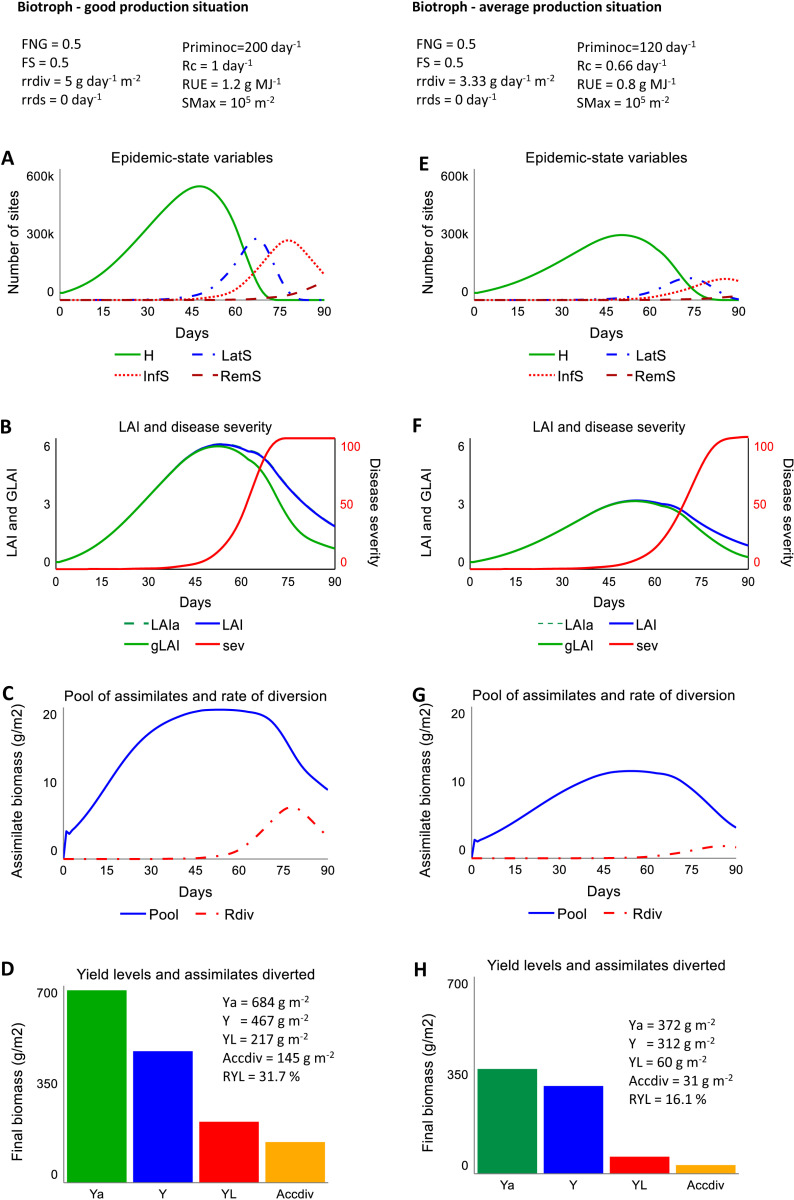
Simulated outputs from DYNAMO-A under the scenarios considering a biotrophic pathogen ideotype. Left panel: outputs in a scenario of good production situation (attainable yield: 684 g.m^-2^ or 6.84 t.ha^-1^); right panel: outputs for a scenario of average production situation (attainable yield: 372 g.m^-2^ or 3.72 t.ha^-1^). Scenario-dependent parameters are given at the top of the figure. A, E: number of healthy (H), latent (LatS), infectious (InfS) and removed (RemS) sites. B, F: LAI: LAI including healthy and diseased leaf areas; gLAI: green LAI; LAIa: attainable (un-injured) LAI (curves for LAIa and LAI are identical); sev: disease severity expressed as the percent of occupied sites. C, G: Pool: pool of assimilates; Rdiv: rate of diversion of assimilates. D, H: Ya: attainable yield, Y: actual yield; YL: yield loss; RYL: relative yield loss; Accdiv: sum of the biomass of assimilates diverted over the simulation. See Table 1 for the meaning of acronyms, and Table 2 for all parameters used to produce the model outputs.

The scenario of a biotrophic pathogen affecting a crop in a good production situation displays an increase of the number of healthy sites together with the LAI, reaches a plateau of about 600,000 sites per m^2^, and then declines because of the combined effects of physiological senescence and disease saturation (Fig 2A,B). The increase in number of latent sites becomes visible at about 40 days. The number of latent sites increases from infection according to a sigmoid shape, and then declines as latent sites become infectious, and as infection decreases because of the decline in the host carrying capacity. The dynamics of infectious sites is similar to that of latent sites, with a delay corresponding to the latent period. The removed sites accumulate towards the end of the crop cycle, when infectious sites become removed. The combined dynamics of the different categories of sites (Fig 2A) lead to a disease progress curve (displayed as severity, Fig 2B) with a sigmoid shape reaching a maximum value of 100%, when all sites are occupied. The [actual] LAI (blue curve: includes healthy and diseased leaf areas) increases until flowering, reaches a maximum of about 6.0, and then declines because of physiological senescence. The green LAI (green curve, includes only green leaf area) dynamics displays a shape similar to that of the LAI, but decreases more strongly towards the end of the crop cycle, as disease severity increases. The attainable LAI dynamics (dashed blue line, hidden by the blue line) is identical to that of the LAI. The dynamics of the rate of diversion (Fig 2C) mirrors the dynamics of infectious sites (Fig 2A): carbohydrate diversion increases, reaches a plateau (of about 5 g day^-1^), and then decreases towards the end of the crop cycle. As a result, the pool of assimilates decreases sharply when the rate of diversion increases (Fig 2C). A good production situation corresponds to an actual yield of 467 g m^-2^, that is, 4.67 t ha^-1^. In this scenario, the biotroph ideotype leads to a yield loss of 217 g m^-2^, and a relative yield loss of 31.7% through light stealing and assimilate diversion (Fig 2D). The accumulated biomass of assimilates diverted by the biotroph is 145 g m^-2^, that is, 67% of the yield which is lost from the biotrophic pathogen.

Compared to the first scenario described above, the scenario considering a biotroph under an average production situation results in dynamics with similar shapes. However, lower numbers of healthy and diseased sites (Fig 2E), a reduced speed of epidemics (Fig 2E, F), reduced *LAI* and *gLAI* (Fig 2F), and reduced pool of assimilates and of their diversion (Fig 2G) are simulated. In other words, both the crop growth and the plant disease epidemics are reduced. The net outcome is an actual yield of 312 g m^-2^, a yield loss of 60 g m^-2^, and a relative yield loss of 16.1% (Fig 2G). The accumulated biomass of assimilates diverted by the biotroph represents 52% of the yield lost from the biotrophic pathogen (Fig 2G).

### 3.2. Simulated epidemics and crop growth for a necrotroph in two production situations

**Fig 3:**
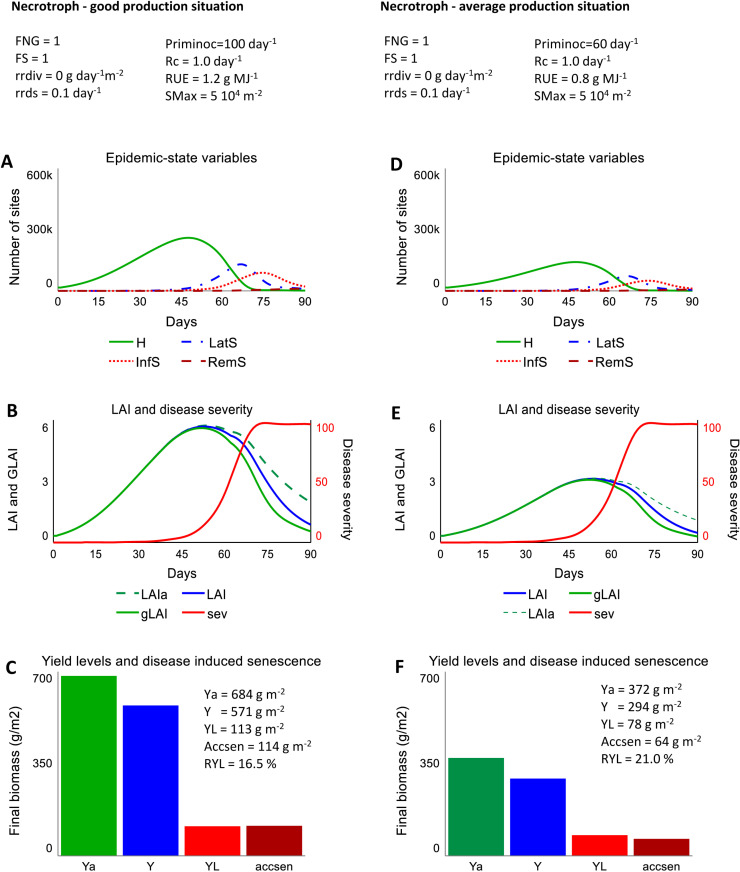
Simulated outputs from DYNAMO-A under the scenarios considering a necrotrophic pathogen ideotype. Left panel: outputs in a scenario of good production situation (attainable yield: 684 g.m^-2^ or 6.84 t.ha^-1^); right panel: outputs for a scenario of average production situation (attainable yield: 372 g.m^-2^ or 3.72 t.ha^-1^). Scenario-dependent parameters are given at the top of the figure A, D: number of healthy (H), latent (LatS), infectious (InfS) and removed (RemS) sites.

The scenario involving the necrotrophic pathogen ideotype affecting a crop grown in a good production situation results in a number of healthy sites increasing, then reaching a plateau at about 300,000 sites, and finally decreasing until no healthy sites remain at day 73 (Fig 3A). The bell-shaped dynamics of latent sites is followed by a similar, although depressed, dynamics of infectious sites (Fig 3A). The LAI (blue curve, including diseased and healthy areas) increases until flowering, and then decreases because of physiological senescence and disease-induced senescence (Fig 3B). The difference between the (actual, diseased) LAI and the attainable LAI (green dashed line) corresponds to the leaf biomass lost because of disease-induced leaf senescence. Furthermore, the difference between the LAI and the green LAI (green curve) corresponds to the LAI occupied by lesions. Disease severity increases according to a sigmoid shape and reaches its maximum (100%) at 73 days (Fig 3B). The effects on crop growth of light stealing and leaf senescence acceleration from the necrotrophic pathogen ideotype result in a yield loss of 113 g m^-2^, and is equal to the accumulated biomass of leaf senesced because of disease (Fig 3C). Similar dynamics are obtained in the scenario where the crop is grown under an average production situation, although with reduced number of sites and LAI (Fig 3 D,E). This last scenario results in a yield loss of 78 g m^-2^, and accumulated biomass of leaf senesced because of disease of 64 g m^-2^ (Fig 3F).

## 4. Discussion

Crop growth models were primarily developed to analyse crop growth under *determining* (radiation, temperature, genotype) and *limiting* factors (water and nutrient supplies; [70]. Very few models have been expanded to consider the effects of r*educing* factors (diseases, animal pests, and weeds). There may be several non-exclusive reasons for that. A first reason relates to disciplinary gaps; a second reason entails the process of model development [71], whereby increasing details are added, rendering further expansion very challenging [20]. Yet progress has been made in recent years with respect to generic model coupling [37,41], although key damage mechanisms were not considered. Multidisciplinary research is necessary to make further progress in that direction, especially if crops and their diseases are to properly be accounted for in climate change analyses. Calls to fill this gap have been reiterated by crop modellers and economists [72–76]. The current work aims to contributing such and effort, with a new generic coupled model, DYNAMO-A, which includes damage mechanisms which have major impact on yield losses, especially in the case of leaf diseases [12, 13]: assimilate diversion, leaf senescence acceleration, as well as light stealing and RUE reduction. We discuss below the hypotheses and implications involved in the coupling so that the approach implemented in DYNAMO-A can be applied in a range of crop growth models.

### 4.1. Assessing model hypotheses and parameterisation

DYNAMO-A is a generic model which represents the two-way processes which are involved when epidemics develop in growing crop stands: the epidemiological processes, which are affected by the crop through the host carrying capacity, and the crop physiological processes, which are affected by the epidemics according to four damage mechanisms. The novelty of this model resides in the two-way coupling of crop growth and plant disease epidemics including four damage mechanisms, represented in a transparent and generic way. Here, DYNAMO-A has been used to analyse the dynamic interactions between pathogen and host populations in scenarios considering ideotypes of a biotrophic pathogen ideotype or of a necrotrophic pathogen affecting a crop grown under good or average production situations. The focus of the current work was to investigate processes related to the interactions between disease and host, and not on the effect of climate or other factors on these interactions. This work, therefore, is intended to present DYNAMO-A as a modelling platform for future use. DYNAMO-A entails a series of hypotheses. Only a few of them are revisited here.

Plant disease epidemiological models generally compute the rate of infection as density-dependent, i.e., proportional to the number of healthy individuals (or sites), or as frequency-dependent, i.e., proportional to the fraction of healthy individuals [77]. In the case of plant diseases caused by pathogens that are aerially dispersed, the rate of infection may increase with the number of sites until canopy closure, because the crop canopy intercepts more propagules as the canopy becomes denser [33,77]. As a result, processes underlying epidemics may switch from density-dependence to frequency-dependence as the foliage (LAI) expands. However, quantitative relationships between LAI and the fraction of spores deposited on the canopy have been very poorly documented. For the sake of simplicity, the rate of infection is made proportional to the fraction of sites available for infection (*COFR*) in DYNAMO-A. Similar outputs to those displayed in Fig 2 were obtained under the assumption of *Rc* being density-dependent at low *LAI* (*Rcv* =  *Rc* x 0.4 x *LAI* for *LAI*<=3, *Rcv* =  *Rc* x 0.4 x 3 for *LAI* > 3; S2 Fig, S3 Fig). This indicates that the outcomes from the analyses reported here may not be affected by modifying the rate of infection. This important hypothesis needs however be revisited when using DYNAMO-A in the future, for example when analysing variations of *Rc* over time.

The effect of the host carrying capacity on the infection rate may furthermore be affected by the spatial dispersal of propagules, and by the spatial structure of epidemics. Disease gradients are expected to be steeper at larger LAI because propagules are intercepted within shorter distances from their source by neighbouring tissues [78, 79]. Steeper disease gradients in turn lead to a decrease in epidemic speed, especially when the primary inoculum is aggregated (e.g., [80]. Again, for the sake of simplicity, this effect was not accounted for in the present work. This hypothesis should be revisited when applying the model in future analyses.

Site size (*SizeS*), the maximum number of sites per area of leaf (*SMax*), and the fraction of non-green area in a site (*FNG*) are three parameters determining the dynamics of epidemics and the green LAI that remains to enable crop growth. These parameters were set according to literature survey, and need to be revised when applying DYNAMO-A to a specific disease.

The rate of crop growth depends on the *RUE*, radiation, *LAI*, and *k* (equation 1). Among them, the parameter *k* is often held at constant, reference values in crop growth models, as in the present work. This parameter however depends on the plant architecture, and therefore can vary with crop development and according to varieties (e.g., [81, 82]). Such variations are often overlooked in crop growth models, despite its important effect on the rate of crop growth.

The intrinsic rates of damage mechanisms (relative rates of assimilate diversion and of disease-induced leaf senescence) have been seldom estimated. Furthermore, their variation according to the conditions of crop growth have virtually not been addressed, despite the possible important impacts on yield losses. Such quantifications are needed in order to estimate the impacts of diseases in various crop growth environments.

### 4.2. Interpretation and insights from the simulated scenarios

The dynamics and outcomes from the simulated scenarios (Figs 2 and 3) can be interpreted as follows:

- The dynamics of the number of sites in the different categories (*H*, *LatS*, *InfS*, *RemS*) display similar shapes in the four simulated scenarios (Figs 2A, 2E and 3A, 3D). This is because the rate of infection is expressed identically in the four scenarios (equation 3).
- The maximum number of healthy sites is reached in all scenarios when the LAI is at its highest level, at about 6. This number is 600,000 in the biotroph scenario (6 x *SMax* =  6 x 100,000) and 300,000 sites (6 x *SMax* =  6 x 50,000) for the scenario involving necrotrophs. As a consequence, all other site categories are about twice lower in the necrotroph than in the biotroph scenario. *SMax* is therefore a very important attribute determining the epidemic magnitude.
- The [actual] simulated LAI is identical to the attainable LAI in the scenario of biotroph ideotype under both good and average production situations (Fig 2B, F). This can be explained because: (1) light stealing and assimilate diversion do not directly affect the LAI (which includes both healthy and diseased tissues), and (2) disease levels start to be high only after flowering, when assimilates are not translocated anymore to leaves, therefore preventing the decrease of photosynthesis (from light stealing and assimilated diversion) to impact the leaf biomass, and therefore the actual (diseased) LAI. On the contrary, both scenarios involving the necrotrophic pathogen ideotype lead to actual LAI values that are lower than the attainable LAI (Fig 3B, E), because diseased and healthy leaf areas are decreased as a consequence of the loss in leaf biomass from disease-induced leaf senescence. This reduction in turn further reduces the host carrying capacity for the epidemic to develop. The rate of infection, the numbers of diseased sites (especially infectious removed sites), and the speed of epidemics are therefore lower in scenarios involving a necrotrophic pathogen ideotype than a biotrophic pathogen ideotype.
- Disease severity displays a sigmoid shape with a maximum of 100% of occupied sites in all simulated scenarios (Figs 2B, F; 3B, E). This sigmoid shape results from the dynamics of the different site categories. Disease severity (the proportion of sites that are diseased in the standing canopy) may not always represent the best descriptor of disease epidemics in the case of necrotrophic pathogens. This is because disease severity in the crop canopy does not account for diseased sites which have been shed by disease-induced senescence. That is why it has been recommended to use the green leaf area and its area under progress curve instead of disease severity to study epidemics of diseases causing defoliation [83–85].
- Yield levels derived from the four scenarios indicate that yield losses are largely determined by assimilate diversion and disease-induced leaf senescence in the case of the biotrophic and necrotrophic pathogen ideotypes, respectively (Figs. 2D, H; 3 C, F). The different values of the relative rate of diversion (*rrdiv*) and of the intrinsic rate of infection (*Rc*), according to varying production situations (and attainable yield), result in larger relative yield losses in the good than in the average production situation (31.7 vs 16.1%). Such a trend has been observed from field experiments in the case of groundnut rust [86] and wheat leaf rust [87]. In the case of the necrotrophic pathogen ideotype, *Rc* and the relative rate of disease-induced senescence were identical for the good and average production situations. The relative yield losses, however, were larger in the average than in the good production situation (21.0 vs 16.5%). This difference can be explained by the lower LAI in the average production situation, leading to larger effects of LAI reduction on radiation interception, because of Beer’s law (*RI* varies less according to LAI when LAI is higher; equation 1). Lower relative yield losses at lower attainable yields have been documented from experiments in the case of wheat septoria tritici blotch [88].
- The overall results thus conform with the literature. This in part reflects the fact that DYNAMO-A builds upon classic concepts of epidemiology and agrophysiology, despite being expressed in the simplest possible way. The selection of simulation runs reported here suggests confidence in the modelling structure which has been designed.

### 4.3. Research Perspectives

This work allowed revisiting classic hypotheses and the need to pursue research on the relation between the rate of infection, the host carrying capacity and propagule dispersal patterns. This work further highlights the need for experimental quantification of interactions between limiting factors (nutrients, water) and damage mechanisms in order to provide a better understanding and more realistic estimates of yield losses, especially when addressing crops grown under a range of limiting factors (i.e., a range of production situations, [58]). For example, drought may increase the impact of rusts on the plant water balance and its physiology [89]. Alternately, an early epidemic of stripe rust generates wheat vulnerability to drought, because it is at the early stage of crop development that root systems are established [90]. Much work is still needed along different angles. Five are briefly considered below in the perspective of a shareable, parameter-sparse, modelling structure.

- Diseases caused by vector-borne, seed-borne, soil-borne, or canopy-borne pathogens [22,91,92] cannot be modelled using the same modelling structure. This diversity of life-styles – not to mention crop “pests” as whole, including nematodes, mites and insects, or parasitic weeds – probably is one root cause of slow progress in the modelling of crop losses. It is, however, our belief that this obstacle may be overcome, at least to some degree. Alternatively, it could be that disease cycles need to be addressed in a much simpler way than what has been done in this study. This would enable disease models to be developed and shared more rapidly. Doing so, however, could be risky and extraordinarily difficult.
- Similarly, crops differ profoundly in their physiology. It would seem, however, that much is already known on the physiological functioning of several world’s key crops, which will facilitate progress.
- Driving functions of models are typically thought of only from the physical, not the biological, standpoint [31]. A once much-studied and well documented area of research is that of the phylloflora. The leaf surface has long been known to host a wide diversity of micro-organisms that interact with pathogens during the infection stage [93–95]. The effects of the phylloflora can be very strong, from preventing an epidemic entirely, to doubling its speed if destroyed, for example by chemicals. Today, research on the phytobiome grows rapidly [96]. It needs to be incorporated in a view that would not ignore this potentially crucial element.
- Spatial aggregation of disease (e.g., [97]), of crop plants (e.g., [98]), and their consequences on disease [99] are tightly linked with the ways pathogens spread, which, in human-made systems, may depend on human activity as well as pathogen biology. Again, the notion of production situation and crop management comes into play in the choices that will have to be made in model development. The consequences of spatial characteristics of epidemics, and then on yield losses may be considerable.
- Any development has costs in terms of model complexity, additional hypotheses, and model parameters. Dealing with models with too large a number of non-measured, sometimes, numerically optimised parameters is in our view dangerous. Sensitivity analysis (e.g., [100]), a technique which is derived from civil engineering, was long seen a solution to handling numerous parameters, many of which numerically optimised. The approach encounters however serious limits as soon as parameters interact among themselves. Our view is that (1) the number of parameters retained should be as small as feasible, (2) parameters should, as much as possible, result from actual observations and experimental measurements, which (3) would consider biological processes occurring at the same level of integration [58]. Following these principles would greatly facilitate model evaluation [101].

In its present state, however, we believe that the generic and transparent structure of DYNAMO-A can be applied as a working platform on aerial plant diseases and be used for a range of purposes, such as phenotyping host plant resistance according to yield gained from components of quantitative resistance, or as estimating plant disease epidemic levels, yield loss, and yield gains from disease management in a range of scenarios of climate change.

## Supporting information

S1 Fig Detailed flowchart of DYNAMO-A. (PDF)

S1 Table Description of the key variables used in DYNAMO-A. (PDF)

S1 Code Code of DYNAMO‐A. (PDF)

S2 Fig Simulated outputs from DYNAMO-A under the scenarios considering a biotrophic pathogen ideotype, with variable Rc. (PDF)

S3 Fig Simulated outputs from DYNAMO-A under the scenarios considering a necrotrophic pathogen ideotype, with variable Rc. (PDF)

## References

[1] Berger RD, Jones JW. A general model for disease progress with functions for variable latency and lesion expansion on growing host plants. Phytopathology. 1985;75(7):792. doi: 10.1094/phyto-75-792

[2] Waggoner PE. Microclimate and plant disease. Annu Rev Phytopathol. 1965;3(1):103–26.

[3] Madden LV. Botanical epidemiology: some key advances and its continuing role in disease management. Eur J Plant Pathol. 2006;115(1):3–23. doi: 10.1007/s10658-005-1229-5

[4] Turechek WW, McRoberts N. Considerations of scale in the analysis of spatial pattern of plant disease epidemics. Annu Rev Phytopathol. 2013;51:453–72. doi: 10.1146/annurev-phyto-081211-173017 23725469

[5] Willocquet L, Fernandez L, Savary S. Effect of various crop establishment methods practised by Asian farmers on epidemics of rice sheath blight caused by *Rhizoctonia solani*. Plant Pathol. 2000;49(3):346–54. doi: 10.1046/j.1365-3059.2000.00454.x

[6] Harris M, Frederiksen R. Concepts and methods regarding host plant resistance to arthropods and pathogens. Annu Rev Phytopathol. 1984;22:247–72.

[7] Boote KJ. Coupling pests to crop growth simulators to predict yield reductions. Phytopathology. 1983;73(11):1581. doi: 10.1094/phyto-73-1581

[8] Rabbinge R, Rijsdijk FH. Disease and crop physiology: a modeller’s point of view. In: Effects of Disease on the Physiology of the Growing Plant. Cambridge, UK: Cambridge University Press; 1981. p. 201–20.

[9] Rabbinge R, Vereyken P. The effects of diseases or pests upon host. Z Pflanzenkrankh Pflanzensch. 1980;87:409–22.

[10] Savary S, Willocquet L. Modeling the Impact of crop diseases on global food security. Annu Rev Phytopathol. 2020;58:313–41. doi: 10.1146/annurev-phyto-010820-012856 32511041

[11] Johnson K. Defoliation, disease, and growth: a reply. Phytopathology. 1987;77:1495–7.

[12] Ayres PG. Effects of disease on the physiology of the growing plant. Cambridge, UK: Cambridge University Press; 1981.

[13] Ayres PG. Pests and pathogens: plant responses to foliar attack. Oxford, UK: Bios Scientific Publishers Limited; 1992.

[14] Strange RN, Scott PR. Plant disease: a threat to global food security. Annu Rev Phytopathol. 2005;43:83–116. doi: 10.1146/annurev.phyto.43.113004.133839 16078878

[15] Acuña I, Andrade-Piedra J, Andrivon D, Armengol J, Arnold AE, Avelino J, et al. A global assessment of the state of plant health. Plant Dis. 2023;107(12):3649–65. doi: 10.1094/PDIS-01-23-0166-FE 37172970

[16] Teng PS. Crop loss assessment and pest management. St Paul, Minn.: APS Press; 1987.

[17] Savary S, McRoberts N, Esker PD, Willocquet L, Teng PS. Production situations as drivers of crop health: evidence and implications. Plant Pathol. 2017;66(6):867–76. doi: 10.1111/ppa.12659

[18] Chakraborty S, Newton AC. Climate change, plant diseases and food security: an overview. Plant Pathol. 2011;60(1):2–14. doi: 10.1111/j.1365-3059.2010.02411.x

[19] Savary S, Willocquet L, Pethybridge SJ, Esker P, McRoberts N, Nelson A. The global burden of pathogens and pests on major food crops. Nat Ecol Evol. 2019;3(3):430–9. doi: 10.1038/s41559-018-0793-y 30718852

[20] Jones JW, Antle JM, Basso B, Boote KJ, Conant RT, Foster I, et al. Brief history of agricultural systems modeling. Agric Syst. 2017;155:240–54. doi: 10.1016/j.agsy.2016.05.014 28701816 PMC5485640

[21] González-Domínguez E, Caffi T, Rossi V, Salotti I, Fedele G. Plant disease models and forecasting: changes in principles and applications over the last 50 Years. Phytopathology. 2023;113(4):678–93. doi: 10.1094/PHYTO-10-22-0362-KD 36624723

[22] Savary S, Nelson A, Djurle A, Esker P, Sparks A, Amorim L. Concepts, approaches, and avenues for modelling crop health and crop losses. Eur J Agron. 2018;100(1):4–18.

[23] Zadoks JC. Systems analysis and the dynamics of epidemics. Phytopathology. 1971;61(6):600–10. 10.1094/Phyto-61-600 40934425

[24] Bove F, Savary S, Willocquet L, Rossi V. Designing a modelling structure for the grapevine downy mildew pathosystem. Eur J Plant Pathol. 2020;157(2):251–68. doi: 10.1007/s10658-020-01974-2

[25] Pfender WF, Upper D. A simulation model for epidemics of stem rust in ryegrass seed crops. Phytopathology. 2015;105(1):45–56. doi: 10.1094/PHYTO-03-14-0068-R 25098493

[26] Rossi V, Racca P, Giosue’ S, Pancaldi D, Alberti I. A simulation model for the development of brown rust epidemics in winter wheat. Eur J Plant Pathol. 1997;103:453–65.

[27] Rossi V, Giosuè S. A dynamic simulation model for powdery mildew epidemics on winter wheat. EPPO Bull. 2003;33(3):389–96. doi: 10.1111/j.1365-2338.2003.00662.x

[28] Savary S, Nelson A, Willocquet L, Pangga I, Aunario J. Modeling and mapping potential epidemics of rice diseases globally. Crop Prot. 2012;34:6–17. doi: 10.1016/j.cropro.2011.11.009

[29] Savary S, Stetkiewicz S, Brun F, Willocquet L. Modelling and mapping potential epidemics of wheat diseases—examples on leaf rust and Septoria tritici blotch using EPIWHEAT. Eur J Plant Pathol. 2015;142(4):771–90. doi: 10.1007/s10658-015-0650-7

[30] Chiarappa L. Crop loss assessment methods. FAO manual on the evaluation and prevention of losses by pests, disease and weeds. Farnham Royal, UK: FAO – Commonwealth Agricultural Bureau; 1971.

[31] Zadoks J, Schein R. Epidemiology and Plant Disease Management. New York, USA: Oxford University Press; 1979.

[32] Johnson KB, Teng PS. Coupling a disease progress model for early blight to a model of potato growth. Phytopathology. 1990;80(4):416. doi: 10.1094/phyto-80-416

[33] Savary S, De Jong P, Rabbinge R, Zadoks J. Dynamic simulation of groundnut rust: a preliminary model. Agric Syst. 1990;32(2):113–41.

[34] Djurle A, Yuen J. A simulation model for *Septoria nodorum* in winter wheat. Agric Syst. 1991;37(2):193–218.

[35] Pacilly F, Hofstede G, van Bueren E, Kessel G, Groot J. Simulating crop-disease interactions in agricultural landscapes to analyse the effectiveness of host resistance in disease control: The case of potato late blight. Ecol Model. 2018;378:1–12.

[36] van Oijen M. Evaluation of breeding strategies for resistance and tolerance to late blight in potato by means of simulation. Neth J Plant Pathol. 1992;98(1):3–11. doi: 10.1007/bf01998073

[37] Bregaglio S, Donatelli M. A set of software components for the simulation of plant airborne diseases. Environ Model Softw. 2015:1–9.

[38] Bregaglio S, Titone P, Cappelli G, Tamborini L, Mongiano G, Confalonieri R. Coupling a generic disease model to the WARM rice simulator to assess leaf and panicle blast impacts in a temperate climate. Eur J Agron. 2016;76:107–17.

[39] Luo Y, Teng P, Fabellar N, TeBeest D. A rice-leaf blast combined model for simulation of epidemics and yield loss. Agric Syst. 1997;53(1):27–39.

[40] Caubel J, Launay M, Ripoche D, Gouache D, Buis S, Huard F. Climate change effects on leaf rust of wheat: Implementing a coupled crop-disease model in a French regional application. Eur J Agron. 2017;90:53–66.

[41] Caubel J, Launay M, Lannou C, Brisson N. Generic response functions to simulate climate-based processes in models for the development of airborne fungal crop pathogens. Ecol Model. 2012;242:92–104.

[42] Willocquet L, Elazegui FA, Castilla N, Fernandez L, Fischer KS, Peng S, et al. Research priorities for rice pest management in tropical Asia: a simulation analysis of yield losses and management efficiencies. Phytopathology. 2004;94(7):672–82. doi: 10.1094/PHYTO.2004.94.7.672 18943898

[43] Willocquet L, Aubertot JN, Lebard S, Robert C, Lannou C, Savary S. Simulating multiple pest damage in varying winter wheat production situations. Field Crop Res. 2008;107(1):12–28. doi: 10.1016/j.fcr.2007.12.013

[44] Savary S, Willocquet L. Simulation modeling in botanical epidemiology and crop loss analysis. The plant health instructor [Internet]. 2014;173. Available from: https://www.apsnet.org/edcenter/disimpactmngmnt/topc/BotanicalEpidemiology/Documents/Downloads/CompleteSimulationModel.pdf

[45] Trivedi P, Leach JE, Tringe SG, Sa T, Singh BK. Plant-microbiome interactions: from community assembly to plant health. Nat Rev Microbiol. 2020;18(11):607–21. doi: 10.1038/s41579-020-0412-1 32788714

[46] Penning de Vries F, Jansen DM, Ten Berge HFM, Bakema A. Simulation of ecophysiological processes of growth in several annual crops. IRRI, Los Baños and PUDOC, Wageningen; 1989.

[47] Kropff MJ, Laar H van, Matthews RB. ORYZA1: An ecophysiological model for irrigated rice production. DLO-Research Institute for Agrobiology and Soil Fertility, Wageningen; WAU-Department of Theoretical Production Ecology, Wageningen; International Rice Research Institute, Los Baños.; 1994.

[48] France J, Thornley JH. Mathematical models in agriculture. Wallingford, UK: CABI; 1984.

[49] Monteith JL. Climate and the efficiency of crop production in Britain. Phil Trans R Soc Lond B. 1977;281(980):277–94.

[50] Van der Plank JE. Plant diseases: epidemics and control. New York, USA: Academic Press; 1963.

[51] Peterson R, Campbell A, Hannah A. A diagrammatical scale for estimating rust severity on leaves and stems of cereals. Can J Res. 1948;26:496–500.

[52] Bastiaans L. Ratio between virtual and visual lesion size as a measure to describe reduction in leaf photosynthesis of rice due to leaf blast. Phytopathology. 1991;81(6):611. doi: 10.1094/phyto-81-611

[53] Kranz J. Comparative epidemiology of plant diseases. New York, USA: Springer; 2003.

[54] Kato H, Kozaka T. Effect of temperature on lesion enlargement and sporulation of *Pyricularia oryzae* in rice leaves. Phytopathology. 1974;64(6):828. doi: 10.1094/phyto-64-828

[55] Tomerlin JR, Eversmeyer MG, Browder LE, Kramer CL. Temperature and host effects on latent and infectious periods and on urediniospore production of *Puccinia recondita* f. sp.*tritici*. Phytopathology. 1983;73(3):414. doi: 10.1094/phyto-73-414

[56] James C. A manual of assessment keys for plant diseases. St. Paul, Minn: APS; 1971. 43 p.

[57] Ou S. Rice Diseases. 2nd edition. Wallingford, UK: CAB International; 1987.

[58] Rabbinge R, Ward SA, Van Laar HH. Simulation and systems management in crop protection. Vol. 32. Wageningen: Pudoc; 1989.

[59] Sinclair TR, Muchow RC. Radiation use efficiency. Adv Agron. 1999;65:215–65.

[60] Roelfs AP, Bushnell WR. The cereal rusts. Volume II. Diseases, distribution, epidemiology, and control. New York: Academic Press; 1985.

[61] Kushalappa AC, Eskes AB. Advances in coffee rust research. Annu Rev Phytopathol. 1989;27(1):503–31.

[62] Yang XB, Royer MH, Tschanz AT, Tsai BY. Analysis and quantification of soybean rust epidemics from seventy-three sequential planting experiments. Phytopathology. 1990;80(12):1421. doi: 10.1094/phyto-80-1421

[63] Primiano IV, Amorim L. Soybean and grapevine rusts accelerate the defoliation rates of host plants. Plant Pathol. 2023;73(3):657–65. doi: 10.1111/ppa.13841

[64] Mendgen K. Nutrient uptake in rust fungi. Phytopathology. 1981;71(9):983. doi: 10.1094/phyto-71-983

[65] Robert C, Bancal M-O, Lannou C. Wheat leaf rust uredospore production on adult plants: influence of leaf nitrogen content and septoria tritici blotch. Phytopathology. 2004;94(7):712–21. doi: 10.1094/PHYTO.2004.94.7.712 18943903

[66] Jensen B, Munk L. Nitrogen‐induced changes in colony density and spore production of *Erysiphe graminis* f.sp. *hordei* on seedlings of six spring barley cultivars. Plant Pathol. 1997;46(2):191–202. doi: 10.1046/j.1365-3059.1997.d01-224.x

[67] Sinclair TR, Horie T. Leaf nitrogen, photosynthesis, and crop radiation use efficiency: a review. Crop Sci. 1989;29(1):90–8. doi: 10.2135/cropsci1989.0011183x002900010023x

[68] Mikaberidze A, Mundt CC, Bonhoeffer S. Invasiveness of plant pathogens depends on the spatial scale of host distribution. Ecol Appl. 2016;26(4):1238–48. doi: 10.1890/15-0807 27509761

[69] Willocquet L, Savary S, Fernandez L, Elazegui F, Teng P. Development and evaluation of a multiple-pest, production situation specific model to simulate yield losses of rice in tropical Asia. Ecol Model. 2000;131:133–59.

[70] Penning de Vries FWT, Laar V. Simulation of plant growth and crop production. Pudoc, Wageningen; 1982.

[71] Penning de Vries F. Phases of development of models. In: Simulation of plant growth and crop production. Wageningen: Pudoc; 1982. p. 20–5.

[72] Donatelli M, Magarey RD, Bregaglio S, Willocquet L, Whish JPM, Savary S. Modelling the impacts of pests and diseases on agricultural systems. Agric Syst. 2017;155:213–24. doi: 10.1016/j.agsy.2017.01.019 28701814 PMC5485649

[73] Jones JW, Antle JM, Basso B, Boote KJ, Conant RT, Foster I, et al. Toward a new generation of agricultural system data, models, and knowledge products: State of agricultural systems science. Agric Syst. 2017;155:269–88. doi: 10.1016/j.agsy.2016.09.021 28701818 PMC5485672

[74] Peng B, Guan K, Tang J, Ainsworth EA, Asseng S, Bernacchi CJ, et al. Towards a multiscale crop modelling framework for climate change adaptation assessment. Nat Plants. 2020;6(4):338–48. doi: 10.1038/s41477-020-0625-3 32296143

[75] Rezaei EE, Webber H, Asseng S, Boote K, Durand JL, Ewert F, et al. Climate change impacts on crop yields. Nat Rev Earth Environ. 2023;4(12):831–46. doi: 10.1038/s43017-023-00491-0

[76] Bregaglio S, Willocquet L, Kersebaum KC, Ferrise R, Stella T, Ferreira TB, et al. Comparing process-based wheat growth models in their simulation of yield losses caused by plant diseases. Field Crop Res. 2021;265:108108. doi: 10.1016/j.fcr.2021.108108

[77] Ferrandino FJ. Effect of crop growth and canopy filtration on the dynamics of plant disease epidemics spread by aerially dispersed spores. Phytopathology. 2008;98(5):492–503. doi: 10.1094/PHYTO-98-5-0492 18943216

[78] Calonnec A, Burie J-B, Langlais M, Guyader S, Saint-Jean S, Sache I, et al. Impacts of plant growth and architecture on pathogen processes and their consequences for epidemic behaviour. Eur J Plant Pathol. 2012;135(3):479–97. doi: 10.1007/s10658-012-0111-5

[79] Savary S, van Santen G. Effect of crop age on primary gradients of late leaf spot (*Cercosporidium personatum*) on groundnut. Plant Pathol. 1992;41(3):265–73. doi: 10.1111/j.1365-3059.1992.tb02348.x

[80] Xu XM, Ridout MS. Effects of initial epidemic conditions, sporulation rate, and spore dispersal gradient on the spatio-temporal dynamics of plant disease epidemics. Phytopathology. 1998;88(10):1000–12. doi: 10.1094/PHYTO.1998.88.10.1000 18944811

[81] Miralles D, Slafer G. Radiation interception and radiation use efficiency of near-isogenic wheat lines with different height. Euphytica. 1997;97:201–8.

[82] Yunusa IAM, Siddique KHM, Belford RK, Karimi MM. Effect of canopy structure on efficiency of radiation interception and use in spring wheat cultivars during the pre-anthesis period in a mediterranean-type environment. Field Crop Res. 1993;35(2):113–22. doi: 10.1016/0378-4290(93)90144-c

[83] Waggoner PE, Berger RD. Defoliation, disease, and growth. Phytopathology. 1987;77(3):393–8.

[84] Filho AB, Carneiro SM, Godoy CV, Amorim L, Berger RD, Hau B. Angular leaf spot of phaseolus beans: relationships between disease, healthy leaf area, and yield. Phytopathology. 1997;87(5):506–15. doi: 10.1094/PHYTO.1997.87.5.506 18945105

[85] Madden LV, Nutter FW Jr. Modeling crop losses at the field scale. Can J Plant Pathol. 1995;17(2):124–37. doi: 10.1080/07060669509500703

[86] Savary S, Zadoks JC. Analysis of crop loss in the multiple pathosystem groundnut-rust-late leaf spot. I. Six experiments. Crop Prot. 1992;11(2):99–109. doi: 10.1016/0261-2194(92)90091-i

[87] Schierenbeck M, Fleitas MC, Simón MR. Nitrogen fertilization and fungicide mixtures in wheat: how do they affect the severity, yield and dynamics of nitrogen under leaf rust infections? Eur J Plant Pathol. 2019;155(4):1061–75. doi: 10.1007/s10658-019-01832-w

[88] Simón MR, Perelló AE, Cordo CA, Struik PC. Influence of *Septoria tritici* on yield, yield components, and test weight of wheat under two nitrogen fertilization conditions. Crop Sci. 2002;42(6):1974–81. doi: 10.2135/cropsci2002.1974

[89] Grimmer MK, John Foulkes M, Paveley ND. Foliar pathogenesis and plant water relations: a review. J Exp Bot. 2012;63(12):4321–31. doi: 10.1093/jxb/ers143 22664583

[90] Hendrix J, Lloyd E. Influence of stripe rust and water stress on wheat roots as revealed in mist culture. Plant Dis Rep. 1970;54:387–9.

[91] Savary S, Willocquet L, Teng PS. Modelling sheath blight epidemics on rice tillers. Agric Syst. 1997;55(3):359–84.

[92] Yang XB, Snow JP, Berggren GT. Analysis of epidemics of Rhizoctonia aerial blight of soybean in Louisiana. Phytopathology. 1990;80(4):386. doi: 10.1094/phyto-80-386

[93] Fokkema NJ. The role of saprophytic fungi in antagonism against *Drechslera sorokiniana* (*Helminthosporium sativum*) on agar plates and on rye leaves with pollen. Physiol Plant Pathol. 1973;3(2):195–205. doi: 10.1016/0048-4059(73)90082-9

[94] Sinha S. Microbiological complex of the phyllosphere and disease control. Indian Phytopathol Soc, Presidential Address. 1965.

[95] Skajennikoff M, Rapilly F. Variations de la phylloflore fongique du blé liées à des traitements fongicides à base de bénomyl1. EPPO Bull. 1981;11(3):295–302. doi: 10.1111/j.1365-2338.1981.tb01936.x

[96] Leach JE, Triplett LR, Argueso CT, Trivedi P. Communication in the Phytobiome. Cell. 2017;169(4):587–96. doi: 10.1016/j.cell.2017.04.025 28475891

[97] Hughes G, Madden LV. Aggregation and incidence of disease. Plant Pathol. 1992;41(6):657–60. doi: 10.1111/j.1365-3059.1992.tb02549.x

[98] Burdon JJ, Chilvers GA. Host density as a factor in plant disease ecology. Annual Rev Phytopathol. 1982;20:143–66.

[99] Hughes G. Incorporating spatial pattern of harmful organisms into crop loss models. Crop Prot. 1996;15(5):407–21. doi: 10.1016/0261-2194(96)00003-8

[100] Confalonieri R, Bellocchi G, Tarantola S, Acutis M, Donatelli M, Genovese G. Sensitivity analysis of the rice model WARM in Europe: exploring the effects of different locations, climates and methods of analysis on model sensitivity to crop parameters. Envir Model Softw. 2010;25(4):479–88. doi: 10.1016/j.envsoft.2009.08.002

[101] Teng P. Validation of computer models of plant disease epidemics: A review of philosophy and methodology. Z Pflanzenkrank Pflanzensch. 1981;49:49–63.

[102] Forrester JW. Industrial dynamics. 1961. Cambridge (Mass.): M.I.T. Press; 1961.

