# Supplementary material for: DYNAMO-A: A generic simulation model coupling crop growth and disease epidemic
Source: PLoS One. 2025 Apr 24;20(4):e0321261. doi: 10.1371/journal.pone.0321261 (PMC12021276; doi:10.1371/journal.pone.0321261)
Supplement: S1 Fig — (PDF) [file pone.0321261.s001.pdf]

DYNAMO-A: a generic simulation model coupling crop growth and disease epidemic

L Willocquet, S Bregaglio, R Ferrise, KH Kim, S Savary

Supporting Information: S1 Figure

Detailed flowchart of DYNAMO-A.
Symbols (state variables: rectangles, rates: valves, flows: double arrows; numerical relations: simple arrows; parameters (fixed or dynamically calculated) and driving functions: circles) used for variables are derived from Forrester (1961).
Some variables have been removed for the sake of legibility.
Dotted circles represent variables copied and pasted to another part of the flowchart.
Symbols in green correspond to variables involved in the host-disease coupling;
Symbols in red correspond to variables involved in the disease-host coupling.
Variables are described in Table 1.

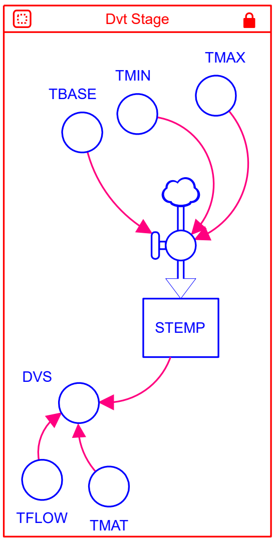
Dvt Stage
TMAX
TMIN
TBASE
STEMP
DVS
TFLOW
TMAT

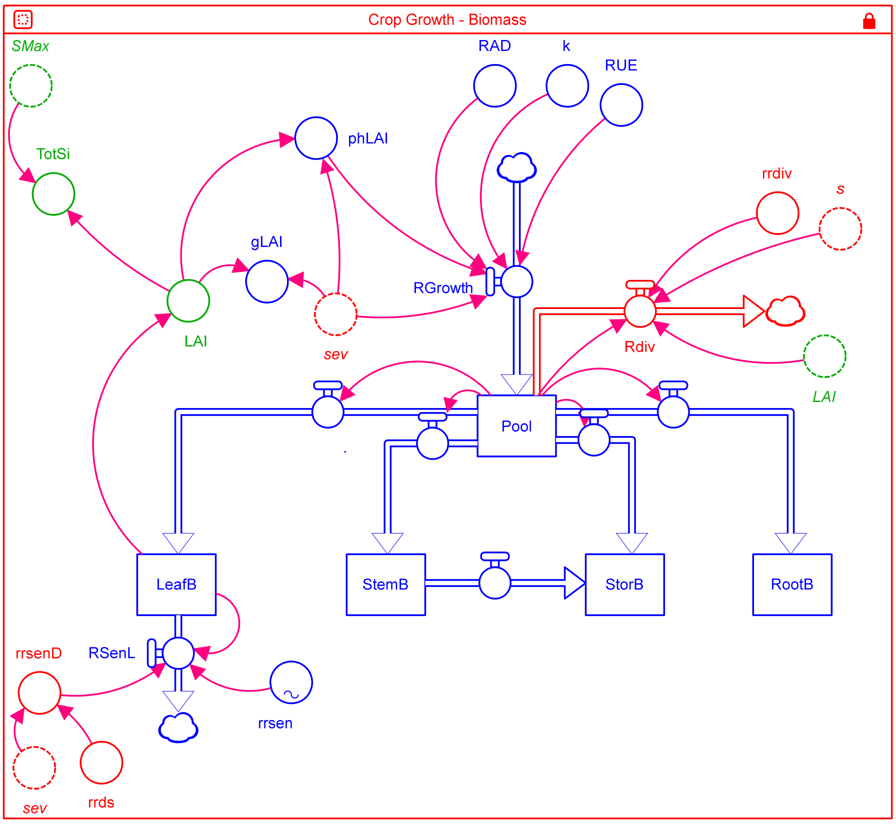
Crop Growth - Biomass
SMax
RAD
k
RUE
TotSi
phLAI
rrdiv
s
gLAI
RGrowth
LAI
sev
Rdiv
LAI
Pool
LeafB
StemB
StorB
RootB
rrsenD
RSenL
rrsen
sev
rrds

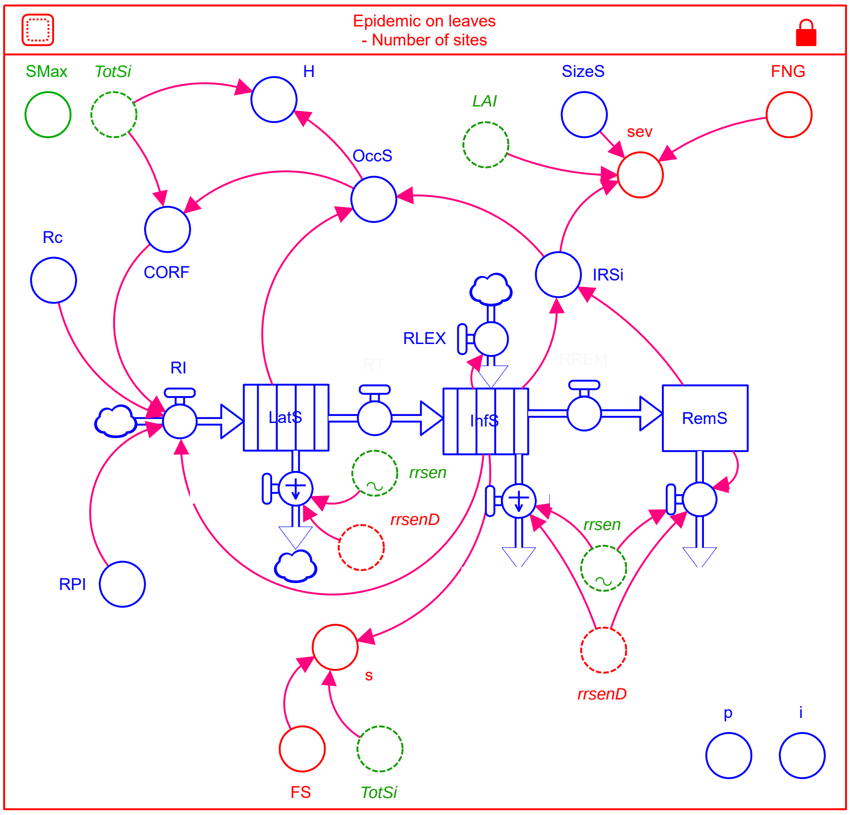
Epidemic on leaves
- Number of sites
SMax
TotSi
H
SizeS
FNG
LAI
sev
OccS
Rc
CORF
IRSi
RLEX
RI
LatS
InfS
RemS
rrsen
rrsenD
rrsen
RPI
s
rrsenD
p
i
FS
TotSi
