# Supplementary material for: DYNAMO-A: A generic simulation model coupling crop growth and disease epidemic
Source: PLoS One. 2025 Apr 24;20(4):e0321261. doi: 10.1371/journal.pone.0321261 (PMC12021276; doi:10.1371/journal.pone.0321261)
Supplement: S1 Table — (PDF) [file pone.0321261.s002.pdf]

DYNAMO-A: a generic simulation model coupling crop growth and disease epidemic

L Willocquet, S Bregaglio, R Ferrise, KH Kim, S Savary

Supporting Information: S1 Table

Description of the key variables used in DYNAMO-A

Acronym	Meaning/computation	Component	Dimension	Unit
**State variables**				
InfS	Number of infectious sites	Epidem	[N]	NSites
LatS	Number of latent sites	Epidem	[N]	NSites
LeafB	Biomass of non-senesced, healthy and diseased leaf blades	Agrophysio	[M]	g
Pool	Pool of assimilates	Agrophysio	[M]	g
RemS	Number of removed sites	Epidem	[N]	N
RootB	Biomass of roots	Agrophysio	[M]	g
StemB	Biomass of stems and leaf sheaths	Agrophysio	[M]	g
STEMP	Sum of temperature above a temperature threshold	Agrophysio	[Θ.T]	°C.day
StorB	Biomass of storage organs	Agrophysio	[M]	g
**Rates**				
RGrowth	Rate of crop growth	Agrophysio	$[M.T^{-1}]$	$g.day^{-1}$
Rdiv	Rate of diversion of assimilates<br>Rdiv = rrdiv*s*LAI	Damage mechanism (E->A)	$[M.T^{-1}]$	$g.day^{-1}$
RI	Rate of infection	Epidem	$[N.T^{-1}]$	$NSites.day^{-1}$
RPI	Rate of primary infection<br>RPI = IF (TIME=onset) THEN PrimInoc ELSE 0	Epidem	$[N.T^{-1}]$	$NSites.day^{-1}$
RLEX	Rate of lesion expansion	Epidem	$[N.T^{-1}]$	$NSites.day^{-1}$
RSenL	Rate of leaf biomass senescence	Agrophysio & Damage mechanism (E->A)	$[M.T^{-1}]$	$g.day^{-1}$
RSenS	Rate of senescence of sites	Epidem & Agrophysio	$[N.T^{-1}]$	$NSites.day^{-1}$
**Parameters**				
β	Ratio of virtual over visible lesion area	Damage mechanism (E->A)	[1]	-
DVS	Development stage – depends on STEMP	Agrophysio	[1]	-
FNG	Fraction of non-green area in infectious or removed sites; typically in the range of 0.4 for cereal rusts on a susceptible variety (Peterson et al, 1948), where a site corresponds to a sporulating area surrounded by green tissue; this fraction can also account for chlorotic area surrounding lesions.	Epidem & Agrophysio	[1]	-
FS	Fraction of the area of an infectious site covered by a sporulating pustule; typically in the range of 0.4 for cereal rusts on a susceptible variety (Peterson et al, 1948), where a site corresponds to a sporulating area surrounded by green tissue.	Epidem & Agrophysio	[1]	-
i	Infectious period	Epidem	[T]	day
k	Coefficient of light extinction	Agrophysio	[1]	-
onset	Day of onset of primary infections	Epidem	[T]	day
p	Latency period	Epidem	[T]	day
PrimInoc	Primary infections	Epidem	$[N.T^{-1}]$	$NSites.day^{-1}$
RAD	Daily radiation	Agrophysio	$[E.T^{-1}]$	$MJ.day^{-1}$
Rc	Intrinsic rate of disease increase	Epidem	$[T^{-1}]$	day^{-1}
RFRUE	Reduction factor for RUE reducer: fraction of RUE reduced per fraction of diseased sites	Damage mechanism (E->A)	[1]	-

rrdiv	Relative rate of diversion of assimilates, per m^2 covered by sporulating diseased tissue	Damage mechanism (E->A)	$[M. L^{-2}.T^{-1}]$	$g.m^{-2}.day^{-1}$
rrds	Relative rate of disease-induced senescence, per disease severity unit	Damage mechanism (E->A)	$[T^{-1}]$	day^{-1}
rrsen	Relative rate of physiological leaf senescence	Agrophysio	$[T^{-1}]$	day^{-1}
RUE	Radiation Use Efficiency	Agrophysio	$[M.E^{-1}]$	$g.MJ^{-1}$
SizeS	Size of a site; in the case of foliar diseases, this represents the area of a leaf which can be occupied (diseased) from infection or lesion expansion. The size of a site can be larger than the visible lesion, as for example in the case of some rusts, for which the site of a site is larger than the size of the pustule	Epidem & Agrophysio	$[L^2]$	m^2
SLA	Specific Leaf Area – depends on DVS	Agrophysio	$[L^2.M^{-1}]$	$m^2.g^{-1}$
SMax	Maximum number of sites which can be occupied on 1 m^2 of host [leaf] tissue; that is, maximum number of lesions; carrying capacity per m^2 of host tissue. For example SMax = 250,000 for wheat leaf rust; SMax = 58,000 for wheat septoria tritici blotch (Savary et al 2015)	Carrying capacity (A->E)	$[N.L^{-2}]$	$NSites.m^{-2}$
TBASE	Threshold temperature for crop development	Agrophysio-driver	[Θ]	°C
TFLOW	Sum of temperature to reach flowering	Agrophysio-driver	[Θ]	°C
TMAT	Sum of temperature to reach maturity	Agrophysio-driver	[Θ]	°C
TMAX	Daily maximum temperature	Agrophysio-driver	[Θ]	°C
TMIN	Daily minimum temperature	Agrophysio-driver	[Θ]	°C
**Intermediate variables**				
CORF	Correction factor<br>CORF = 1-(MIN(1,OccS/TotSi))	Epidem & Agrophysio	[1]	-
H	Number of healthy sites<br>H = MAX(0, TotSi-OccS)	Epidem & Agrophysio	[N]	NSites
IRSi	Number of infectious and removed sites<br>IRSi = InfS+RemS	Epidem	[N]	NSites
LAI	Non senesced, healthy and diseased LAI<br>LAI = MAX(0, LeafB*SLA)	Agrophysio	$[L^2]$	m^2
gLAI	Green LAI<br>MAX(0, LAI*(1-sev))	Agrophysio	$[L^2]$	m^2
OccS	Number of occupied sites<br>OccS = LAtS+InfS+RemS	Epidem	[N]	NSites
phLAI	Photosynthetically active LAI<br>$phLAI = LAI*(1-sev)^{\beta}$	Damage mechanism (E->A)	$[L^2]$	m^2
rrsenD	Relative rate of disease-induced senescence<br>rrsenD = rrds*sev	Damage mechanism (E->A)	$[T^{-1}]$	day^{-1}
s	Fraction of LAI covered by sporulating pustules (on infectious sites)<br>s = FS*(InfSi/TotSi)	Agrophysio & Epidem	[1]	-
sev	Disease severity: fraction of LAI with non-green [leaf] area from infectious and removed sites<br>sev = SizeS*FNG*IRSi/LAI	Agrophysio & Epidem	[1]	-
TotSi	Maximum number of sites which can be occupied, that is, the carrying capacity of host tissue in a crop on 1 m^2 of ground (the system size)<br>TotSi = LAI*SMax<br>TotSi also represents the total number of sites (healthy, latent, infectious and removed)	Carrying capacity (A->E)	[N]	NSites

Dimensions and units consider a system of 1 m^2 of crop.

Epidem = epidemiological component, Agrophysio = agrophysiological component.

A->E: coupling from agrophysiological processes to epidemics; E->A: coupling from epidemics to agrophysiological processes.
