# Supplementary material for: DYNAMO-A: A generic simulation model coupling crop growth and disease epidemic
Source: PLoS One. 2025 Apr 24;20(4):e0321261. doi: 10.1371/journal.pone.0321261 (PMC12021276; doi:10.1371/journal.pone.0321261)
Supplement: S1 Code — (PDF) [file pone.0321261.s003.pdf]

DYNAMO-A: a generic simulation model coupling crop growth and disease epidemic

L Willocquet, S Bregaglio, R Ferrise, KH Kim, S Savary

Supporting Information: S1 Code

Code of DYNAMO-A.

```
Accdiv(t) = Accdiv(t - dt) + (Rdiv) * dt
  INIT Accdiv = 0
  INFLOWS:
    Rdiv = MIN(Pool, rrdiv*s*LAI)
accsenDB(t) = accsenDB(t - dt) + (rsenDB1) * dt
  INIT accsenDB = 0
  INFLOWS:
    rsenDB1 = RsenDB
InfS(t) = InfS(t - dt) + (RT + RLEX - RREM - RsenIS) * dt
  INIT InfS = 0
    TRANSIT TIME = i
  INFLOWS:
    RT = CONVEYOR OUTFLOW
    RLEX = RRLEX*InfS*CORF
  OUTFLOWS:
    RREM = CONVEYOR OUTFLOW
    RsenIS = LEAKAGE OUTFLOW
      LEAKAGE FRACTION = rrsen+rrsenDInfS-(rrsen*rrsenDInfS)
LatS(t) = LatS(t - dt) + (RI - RT - RsenLS) * dt
  INIT LatS = 0
    TRANSIT TIME = p
    CAPACITY = INF
    INFLOW LIMIT = INF
  INFLOWS:
    RI = RPI+(DMFR*InfS*CORF)
  OUTFLOWS:
    RT = CONVEYOR OUTFLOW
    RsenLS = LEAKAGE OUTFLOW
      LEAKAGE FRACTION = rrsen+rrsenD-(rrsen*rrsenD)
LeafB(t) = LeafB(t - dt) + (PartL - RSenL) * dt
  INIT LeafB = 10
  INFLOWS:
    PartL = CPL*(Pool-Rdiv)
  OUTFLOWS:
    RSenL = (rrsen+rrsenD-(rrsen*rrsenD))*LeafB
MaxStemb(t) = MaxStemb(t - dt) + (rmaxstemb) * dt
  INIT MaxStemb = 6
  INFLOWS:
    rmaxstemb = PartS
```

```
Pool(t) = Pool(t - dt) + (RGrowth - PartS - PartL - PartSO - PartR - Rdiv) * dt
   INIT Pool = 0
   INFLOWS:
      RGrowth = RAD*RUE*(1-EXP(-k*phLAI))*(1-(RFRUE*sevNG))
   OUTFLOWS:
      PartS = CPS*(Pool-Rdiv)
      PartL = CPL*(Pool-Rdiv)
      PartSO = CPP*(Pool-Rdiv)
      PartR = CPR*(Pool-Rdiv)
      Rdiv = MIN(Pool, rrdiv*s*LAI)
RemS(t) = RemS(t - dt) + (RREM - RsenRS) * dt
   INIT RemS = 0
   INFLOWS:
      RREM = CONVEYOR OUTFLOW
   OUTFLOWS:
      RsenRS = (rrsen+rrsenD-(rrsen*rrsenD))*RemS
RootB(t) = RootB(t - dt) + (PartR) * dt
   INIT RootB = 5
   INFLOWS:
      PartR = CPR*(Pool-Rdiv)
SInfS(t) = SInfS(t - dt) + (RsenIS) * dt
   INIT SInfS = 0
   INFLOWS:
      RsenIS = LEAKAGE OUTFLOW
         LEAKAGE FRACTION = rrsen+rrsenDInfS-(rrsen*rrsenDInfS)
SRemS(t) = SRemS(t - dt) + (RsenRS) * dt
   INIT SRemS = 0
   INFLOWS:
      RsenRS = (rrsen+rrsenD-(rrsen*rrsenD))*RemS
StemB(t) = StemB(t - dt) + (PartS - RTransloc) * dt
   INIT StemB = 6
   INFLOWS:
      PartS = CPS*(Pool-Rdiv)
   OUTFLOWS:
      RTransloc = IF(DVS>1) THEN ddist ELSE 0
STEMP(t) = STEMP(t - dt) + (Dtemp) * dt
   INIT STEMP = 320
   INFLOWS:
      Dtemp = MAX(0, ((TMAX+TMIN)/2)-TBASE)
StorB(t) = StorB(t - dt) + (PartSO + RTransloc) * dt
   INIT StorB = 0
   INFLOWS:
      PartSO = CPP*(Pool-Rdiv)
      RTransloc = IF(DVS>1) THEN ddist ELSE 0
transloc(t) = transloc(t - dt) + (rtransloccopy) * dt
   INIT transloc = 0
   INFLOWS:
      rtransloccopy = RTransloc
```

ACI = LatS+IRSi
beta1 = 1
CORF = 1-(MIN(1,ACI/TotSi))
CPL = CPPL*(1-CPR)
CPP = CPPP*(1-CPR)
CPPL = GRAPH(DVS)
(0.000, 0.5500), (0.100, 0.5360), (0.200, 0.5210), (0.300, 0.5070), (0.400, 0.4930), (0.500, 0.4790), (0.600, 0.4640), (0.700, 0.4500), (0.800, 0.3000), (0.900, 0.1500), (1.000, 0.0000), (1.100, 0.0000), (1.200, 0.0000), (1.300, 0.0000), (1.400, 0.0000), (1.500, 0.0000), (1.600, 0.0000), (1.700, 0.0000), (1.800, 0.0000), (1.900, 0.0000), (2.000, 0.0000)
CPPP = GRAPH(DVS)
(0.000, 0.000), (0.050, 0.000), (0.100, 0.000), (0.150, 0.000), (0.200, 0.000), (0.250, 0.000), (0.300, 0.000), (0.350, 0.000), (0.400, 0.000), (0.450, 0.000), (0.500, 0.000), (0.550, 0.000), (0.600, 0.000), (0.650, 0.000), (0.700, 0.000), (0.750, 0.000), (0.800, 0.143), (0.850, 0.286), (0.900, 0.429), (0.950, 0.571), (1.000, 0.714), (1.050, 0.857), (1.100, 1.000), (1.150, 1.000), (1.200, 1.000), (1.250, 1.000), (1.300, 1.000), (1.350, 1.000), (1.400, 1.000), (1.450, 1.000), (1.500, 1.000), (1.550, 1.000), (1.600, 1.000), (1.650, 1.000), (1.700, 1.000), (1.750, 1.000), (1.800, 1.000), (1.850, 1.000), (1.900, 1.000), (1.950, 1.000), (2.000, 1.000)
CPR = GRAPH(DVS)
(0.000, 0.300), (0.100, 0.2625), (0.200, 0.225), (0.300, 0.188), (0.400, 0.150), (0.500, 0.112), (0.600, 0.075), (0.700, 0.038), (0.800, 0.000), (0.900, 0.000), (1.000, 0.000), (1.100, 0.000), (1.200, 0.000), (1.300, 0.000), (1.400, 0.000), (1.500, 0.000), (1.600, 0.000), (1.700, 0.000), (1.800, 0.000), (1.900, 0.000), (2.000, 0.000)
CPS = (1-CPL-CPP)*(1-CPR)
ddist = 0.005*MaxStemb
DMFR = 1
DVS = IF STEMP<TFLOW THEN STEMP/TFLOW ELSE 1+((STEMP-TFLOW)/(TMAT-TFLOW))
finalInfremS = InfS+RemS+SInfS+SRemS
FNG = 0.5
FS = 0.5
gLAI = MAX(0, LAI*(1-sevNG))
H = MAX(0, TotSi-ACI)
i = 20
IRSi = InfS+RemS
k = 0.6
LAI = MAX(0, LeafB*SLA)
onset = 10
p = 10
phLAI = LAI*((1-sevNG)^beta1)
PrimInoc = 200
RAD = 17
RFRUE = 0
RPI = IF (TIME=onset) THEN PrimInoc ELSE 0
rrdiv = 5
rrds = 0
RRLEX = 0
rrsen = GRAPH(DVS)

(0.000, 0), (0.100, 0), (0.200, 0), (0.300, 0), (0.400, 0), (0.500, 0), (0.600, 0), (0.700, 0), (0.800, 0), (0.900, 0), (1.000, 0), (1.100, 0.013), (1.200, 0.026), (1.300, 0.04), (1.400, 0.04), (1.500, 0.04), (1.600, 0.04), (1.700, 0.04), (1.800, 0.04), (1.900, 0.04), (2.000, 0.04)
rrsenD = rrds*sevNG
rrsenDInfS = toto*(RemS+InfS)
RsenDB = (rrsenD-(rrsenD*rrsen/2))*LeafB
RUE = 1.2
s = FS*InfS/TotSi
sev = MIN(100, 100*ACI/(LAI*SMax))
sevNG = SizeS*FNG*IRSi/LAI
SizeS = 0.00001
SLA = GRAPH(DVS)
(0.000, 0.037), (1.000, 0.018), (2.000, 0.017)
SLActe = 0.02
SMax = 100000
TBASE = 8
TFLOW = 1500
TMAT = 2000
TMAX = 30
TMIN = 24
toto = rrds*FNG/TotSi
TotSi = LAI*SMax
Y = ENDVAL(StorB)
