# Supplementary material for: DYNAMO-A: A generic simulation model coupling crop growth and disease epidemic
Source: PLoS One. 2025 Apr 24;20(4):e0321261. doi: 10.1371/journal.pone.0321261 (PMC12021276; doi:10.1371/journal.pone.0321261)

DYNAMO-A: a generic simulation model coupling crop growth and disease epidemic

L Willocquet, S Bregaglio, R Ferrise, KH Kim, S Savary

Supporting Information: S2 Figure

Simulated outputs from DYNAMO-A under the scenarios considering a biotrophic pathogen ideotype, with variable Rc.

Left panel: outputs in a scenario of good production situation (attainable yield: 684 $g.m^{-2}$ or 6.84 $t.ha^{-1}$); right panel: outputs for a scenario of average production situation (attainable yield: 372 $g.m^{-2}$ or 3.72 $t.ha^{-1}$).

Scenario-dependent parameters are given at the top of the figure.

A, E: number of healthy (Hp, latent (LatS), infectious (InfS) and removed (RemS) sites.

B, F: LAI: LAI including healthy and diseased leaf areas; gLAI: green LAI; LAIa: attainable (un-injured) LAI (curves for LAIa and LAI are identical); sev: disease severity expressed as the percent of occupied sites.

C, G: Pool: pool of assimilates; Rdiv: rate of diversion of assimilates.

D, H: Ya: attainable yield, Y: actual yield; YL: yield loss; RYL: relative yield loss; Accdiv: sum of the biomass of assimilates diverted over the simulation.

See Table 1 for the meaning of acronyms, and Table 2 for all parameters used to produce the model outputs.

Biotroph - good production situation

FNG = 0.5
FS = 0.5
rrdiv = 5 g day^{-1} m^{-2}
rrds = 0 day^{-1}

Priminoc=200 day^{-1}
Rc = 1 day^{-1}
RUE = 1.2 g MJ^{-1}
SMax = 10^5 m^{-2}

Biotroph - average production situation

FNG = 0.5
FS = 0.5
rrdiv = 3.33 g day^{-1} m^{-2}
rrds = 0 day^{-1}

Priminoc=120 day^{-1}
Rc = 0.66 day^{-1}
RUE = 0.8 g MJ^{-1}
SMax = 10^5 m^{-2}

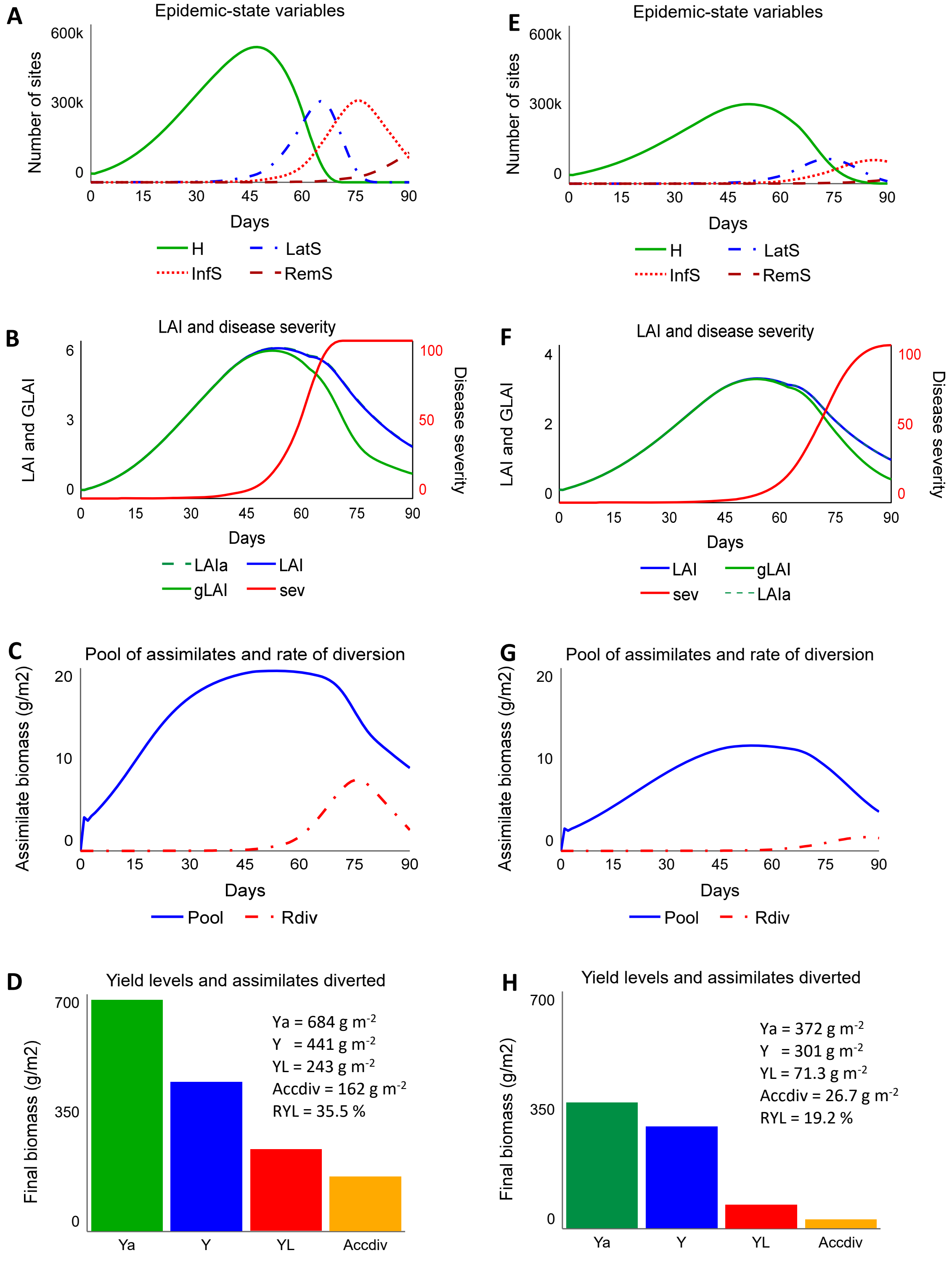

Supplement: S2 Fig — (PDF) [file pone.0321261.s004.pdf]
