# Supplementary material for: DYNAMO-A: A generic simulation model coupling crop growth and disease epidemic
Source: PLoS One. 2025 Apr 24;20(4):e0321261. doi: 10.1371/journal.pone.0321261 (PMC12021276; doi:10.1371/journal.pone.0321261)

DYNAMO-A: a generic simulation model coupling crop growth and disease epidemic

L Willocquet, S Bregaglio, R Ferrise, KH Kim, S Savary

Supporting Information: S3 Figure

Simulated outputs from DYNAMO-A under the scenarios considering a necrotrophic pathogen ideotype, with variable Rc.

Left panel: outputs in a scenario of good production situation (attainable yield: 684 $g.m^{-2}$ or 6.84 $t.ha^{-1}$); right panel: outputs for a scenario of average production situation (attainable yield: 372 $g.m^{-2}$ or 3.72 $t.ha^{-1}$).
Scenario-dependent parameters are given at the top of the figure A, D: number of healthy (H), latent (LatS), infectious (InfS) and removed (RemS) sites.
B, E: LAI: LAI including healthy and diseased leaf areas; gLAI: green LAI; LAIa: attainable (un-injured) LAI; sev: disease severity expressed as the percent of occupied sites.
C, F: Ya: attainable yield, Y: actual yield; YL: yield loss; RYL: relative yield loss; Accsen: sum of the biomass of leaves senesced from disease.
See Table 1 for the meaning of acronyms, and Table 2 for all parameters used to produce the model outputs.

Necrotroph - good production situation

FNG = 1
FS = 1
rrdiv = 0 g day^{-1}m^{-2}

Priminoc=100 day^{-1}
Rc = 1.0 day^{-1}
RUE = 1.2 g MJ^{-1}

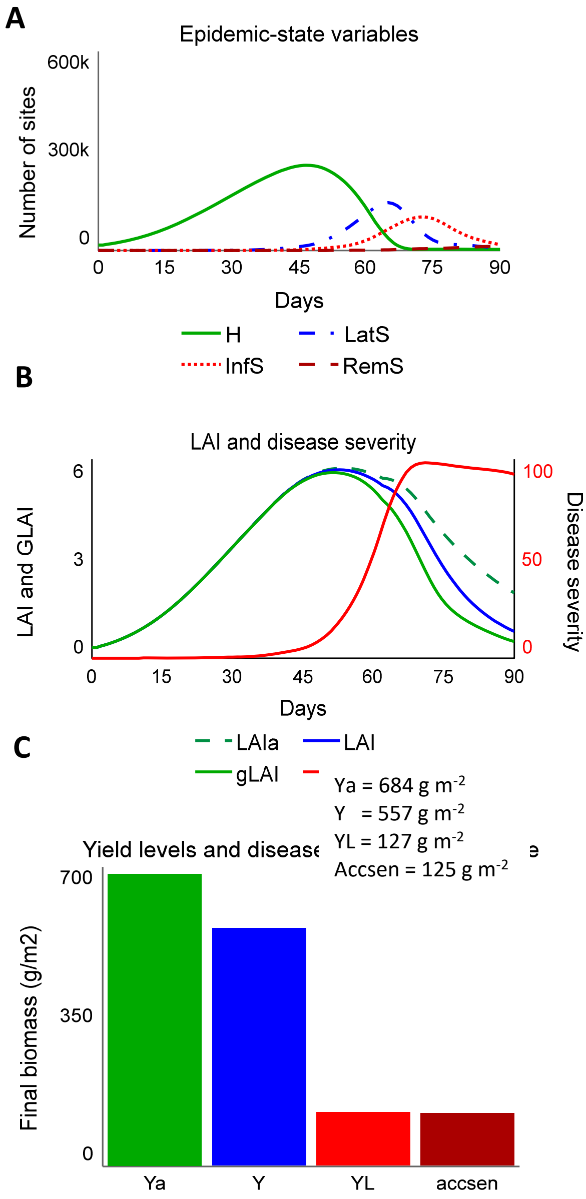

Necrotroph - average production situation

FNG = 1
FS = 1
rrdiv = 0 g day^{-1}m^{-2}

Priminoc=60 day^{-1}
Rc = 1.0 day^{-1}
RUE = 0.8 g MJ^{-1}

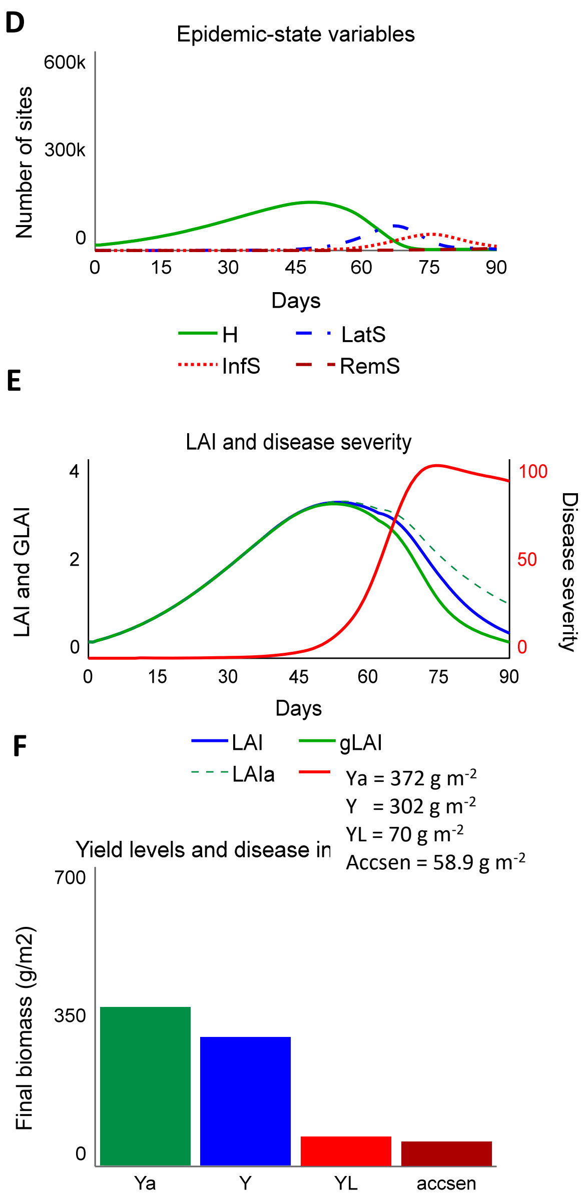

Supplement: S3 Fig — (PDF) [file pone.0321261.s005.pdf]
